# Inflammatory stimulation of astrocytes affects the expression of miRNA-22-3p within NSCs-EVs regulating remyelination by targeting KDM3A

**DOI:** 10.1186/s13287-023-03284-w

**Published:** 2023-03-23

**Authors:** Tianyu Han, Peiwen Song, Zuomeng Wu, Cancan Wang, Yunlei Liu, Wang Ying, Kaixuan Li, Cailiang Shen

**Affiliations:** 1 Department of Orthopedics (Spinal Surgery), The First Affiliated Hospital of Anhui Medical University, 218 Jixi Road, Shushan District, Hefei City, Anhui Province China; 2 Department of Clinical Laboratory, No.2 People’s Hospital of Fuyang, Fuyang city, China; 3 Department of Medical Imaging, The First Affiliated Hospital of Anhui Medical University, Hefei city, China

**Keywords:** Spinal cord injury, Neural stem cells, miRNA-22, Remyelination

## Abstract

**Background:**

Endogenous neural stem cells (NSCs) are critical for the remyelination of axons following spinal cord injury (SCI). Cell–cell communication plays a key role in the regulation of the differentiation of NSCs. Astrocytes act as immune cells that encounter early inflammation, forming a glial barrier to prevent the spread of destructive inflammation following SCI. In addition, the cytokines released from astrocytes participate in the regulation of the differentiation of NSCs. The aim of this study was to investigate the effects of cytokines released from inflammation-stimulated astrocytes on the differentiation of NSCs following SCI and to explore the influence of these cytokines on NSC–NSC communication.

**Results:**

Lipopolysaccharide stimulation of astrocytes increased bone morphogenetic protein 2 (BMP2) release, which not only promoted the differentiation of NSCs into astrocytes and inhibited axon remyelination in SCI lesions but also enriched miRNA-22-3p within extracellular vesicles derived from NSCs. These miRNA-22 molecules function as a feedback loop to promote NSC differentiation into oligodendrocytes and the remyelination of axons following SCI by targeting KDM3A.

**Conclusions:**

This study revealed that by releasing BMP2, astrocytes were able to regulate the differentiation of NSCs and NSC–NSC communication by enriching miRNA-22 within NSC-EVs, which in turn promoted the regeneration and remyelination of axons by targeting the KDM3A/TGF-beta axis and the recovery of neurological outcomes following SCI.

**Supplementary Information:**

The online version contains supplementary material available at 10.1186/s13287-023-03284-w.

## Introduction

Spinal cord injury (SCI) is caused by mechanical forces to the spinal cord, resulting in irreversible nerve cell death [1]. Following primary mechanical trauma, the accumulation of immune cells, free radicals and other cellular debris at the injured lesion site leads to a secondary injury that further kills the adjacent surviving cells and induces demyelination of the axons, which results in a larger lesion, axonal dieback away from the cavity and worse neurological function [1–5]. In an attempt to promote functional recovery, researchers have designed treatments from several different perspectives, ranging from controlling secondary injury, which reduces the apoptosis of surviving nerve cells, to the transplantation of exogenous nerve cells, which aids in axonal regrowth and remyelination, thus rebuilding neural circuits [4–6]. However, these current treatments have achieved limited effects.

The discovery of endogenous neural stem cells (NSCs) in the spinal cord has suggested a promising strategy for the treatment of SCI [7]. NSCs are multipotent and self-renewing stem cells and have the ability to differentiate into mature nerve cells, including neurons, oligodendrocytes and astrocytes [8]. NSCs have been found to exist in all main subdivisions of the adult mammalian central nervous system, including the spinal cord [9–11]. Moreover, further studies have reported that NSCs are activated, migrate, and accumulate at lesion sites following SCI [7, 10]. However, most of these activated endogenous NSCs differentiate into astrocytes rather than oligodendrocytes, which contribute to the remyelination of the surviving axons [7, 12]. Therefore, it is critical to mediate the differentiation of endogenous NSCs, boosting axon regeneration and remyelination.

Inflammation plays a key role in secondary injury following SCI and has been proven to be associated with many pathological alterations of neurotrauma [4, 5, 13]. Astrocytes, as immune cells, rapidly form astrocytic barriers surrounding the injured lesion core, separating the inflammatory cells from adjacent tissues [13, 14]. However, inflammation-stimulated astrocytes might undergo a phenotypic transformation known as reactive astrogliosis accompanied by the alteration of their paracrine signaling, which directly affects the pathology following SCI [15–17]. For example, in response to inflammatory stimulation, astrocytes produce more proinflammatory cytokines, including interleukin-1 alpha (IL-1α) and tumor necrosis factor alpha (TNF-α) [18], which might exacerbate the toxic inflammatory process and lead to neuronal apoptosis. Moreover, upon inflammatory stimulation, astrocytes also release some axon inhibitors, including bone morphogenetic proteins (BMPs) [19–21]. BMPs, known as growth factors, are part of the transforming growth factor beta (TGF-β) superfamily. They have been reported to be potent inhibitors of oligodendroglial lineage specification, directly promoting NSC differentiation into astrocytes. In the early phase of SCI, with the activation of the inflammatory response, BMPs are largely released by immune cells (including astrocytes) and are rapidly upregulated in lesion sites [22, 23]. Therefore, understanding the effects of these cytokines or factors released from inflammation-stimulated astrocytes on the differentiation of NSCs would be crucial to designing a strategy for the treatment of SCI.

Extracellular vesicles (EVs) are membrane-delimited particles that are 30–150 nm and released by nearly all kinds of cells, and emerging evidence has reported that through EVs, endogenous NSCs initiate cell–cell communication with adjacent cells [24, 25], including with NSCs themselves [26]. EVs carry various bioactive cargoes and play a key role in regulating physiological functions and pathophysiological processes in many disease systems [17, 27–34]. EVs derived from NSCs have been reported to promote neurological recovery following neurotrauma due to their anti-inflammatory, neurogenic, and neurotrophic effects [25, 35, 36]. Moreover, EVs, as carriers of miRNAs, proteins, and lipids, can be mediated by external stimuli [27, 37]. Studies have demonstrated that with different external stimulations of cells, EVs can exert distinct biological functions [38, 39]. However, whether EVs derived from NSCs that communicate with inflammatory stimulated astrocytes affect the differentiation of adjacent NSCs remains unclear.

In the present study, we investigated the effects of cytokines released from inflammation-stimulated astrocytes on the differentiation of NSCs following SCI and explored the influence of these cytokines on NSC–NSC communication. We demonstrated that inflammatory stimulation significantly promoted astrocytes to release more BMP2, which directly promoted the differentiation of NSCs into astrocytes, leading to the failure of axon regrowth. Furthermore, we found that the cytokines released from the inflammatory stimulated astrocytes were able to enrich the miRNA-22-3p within NSC-EVs. These EVs were able to mediate the differentiation of NSCs into oligodendrocytes by targeting KMD3A and repressing the TGF-β signaling pathway. Herein, we showed a BMP-miRNA-22-TGF-β regulatory loop, which maintained the glial balance of NSCs to prevent the overformation of astrocytic scars caused by inflammatory stimulation.

## Method

### Culture of astrocytes and collection of the conditioned medium

Primary astrocytes were obtained from the cerebral cortex of postnatal Day 1 SD rats as described in a previous study [40]. The isolated cells were plated on culture flasks at a density of 1 × 10^6^ cells/cm^2^ and adherently cultured for 7 days with DMEM/F12 (Gibco, USA) supplemented with 10% fetal bovine serum (FBS) (Gibco, USA) and 1% antibiotic solution. The medium was changed every three days, and cells were passaged when they reached 90% confluence. Passage 3–6 astrocytes were used for the experiment.

To collect the conditioned medium (CM) from astrocytes, 90% confluent adherent astrocytes were washed with PBS three times and cultured with serum-free DMEM/F12 for 24 h. Next, the cultured serum-free medium was collected, which was designated as primary astrocyte CM (As-CM). Then, the primary As-CMs from different individual flasks were pooled and concentrated at 4000×*g* for 15 min by using 10-kDa MW filter units (Millipore, USA). The collected CM was filtered by a 0.22-μm filter (Millipore, USA) and stored at − 80 °C [41]. To collect the CM from the LPS-stimulated astrocytes, 10 ng/ml LPS was added to 90% confluent adherent astrocytes and cocultured for 12 h. Then, the cells were washed three times with PBS, and the medium was switched to serum-free DMEM/F12 for another 24 h of culture. The culture medium was pooled together as primary LPS-As-CM and concentrated to obtain LPS-AS-CM. The level of BMP2/4/7 was detected by ELISA kits (Invitrogen, USA) following the instructions of the manufacturer.

### NSC culture, transfection, and differentiation

NSCs were isolated from the subventricular zone of SD rats and were suspended as neurospheres in culture medium containing DMEM/F12, 2% B27 (Gibco, USA), 20 ng/ml epidermal growth factor (EGF) (Gibco, USA), and 10 ng/ml basic fibroblast growth factor (bFGF) (Gibco, USA) (details of the method of primary NSC culture are described in our previous studies [42, 43]).

The overexpression or knockdown of miRNA-22-3p in NSCs was performed with the use of 50 nM miRNA-22-3p mimics or inhibitors (Nanjing Kaien, China), nontarget control miRNA mimics (mimics-NC), or a scrambled control sequence (inhibitors-NC) for NCs or anti-NCs. HiPerfect transfection reagent (Hilden, Germany) was used following the manufacturer’s instructions. PCR was used to confirm the effects of miRNA-22-3p mimics and inhibitors in NSCs.

The passage 2 NSCs were dissociated by trypsin and plated on glass coverslips in 10% FBS-DMEM/F12. After 24 h of culture, the medium was switched to differentiation medium (DMEM/F12 containing 2% B27 and 1% antibiotic solution) or differentiation medium with one of the following: As-CM; LPS-As-CM; LPS-As-CM + 200 ng/ml Noggin (Sigma); NSC-EVs; EVs derived from the As-CM-stimulated NSCs (As-NSC-EVs); EVs derived from the LPS-As-CM-stimulated NSCs (LPS-As-NSC-EVs); 50 nM miRNA-22-3p mimics; or 50 nM miRNA-22-3P inhibitors + LPS-As-NSC-EVs. The medium was changed every three days. After 7 days of culture, the cells were subjected to immunohistochemistry staining.

### NSC-EVs collection

The supernatant of 7-day-cultured NSCs was collected. This collected medium was centrifuged at 300×*g* for 10 min, 2000×*g* for 20 min, and finally at 10,000×*g* for 45 min at 4 °C to remove cell debris. Then, the supernatant was centrifugation at 10,0000×*g* for 90 min at 4 °C to collect the NSC-EVs. To identify NSC-EVs, transmission electron microscopy (TEM) and western blot analysis were performed (the antibodies used were as follows: anti-CD 63 1:1000, anti-CD 9 1:2000, and anti-TSG 101 1:1000). (The identification of NSCs-EVs is shown in Additional file 1: Figure S1.) The diameters of the NSC-EVs were assessed by dynamic light scattering. The harvested NSC-EVs were then dissolved in 100 μl of PBS and stored at − 80 °C.

To collect EVs from NSCs stimulated by different situations, NSCs cultured for 7 days were first treated with As-CM, LPS-As-CM with or without 200 ng/ml Noggin, or 20 ng/ml BMP2 for 24 h. Then, the cells and supernatants were collected and concentrated at 800 rpm for 5 min. After three washes with DMEM/F12, the NSCs were resuspended in culture. After 24 h, the supernatants were obtained for concentration to collect the EVs as described above.

### Animal protocols and tissue processing

A direct weight-drop injury to the spinal cord at the T 9–10 level was performed to induce SCI models by using an infinite-horizon spinal cord impactor (IH-0400), and a polyethylene catheter was placed at the injured level for intrathecal injection. (Details of the SCI procedure have been addressed in our previous study [42].) The injured rats received a continuous injection of 25 μl DMEM/F12, 50 μl As-CM, 50 μl LPS-As-CM, 50 μl LPS-As-CM + 200 ng Noggin, 25 μl NSC-EVs, 25 μl As-NSC-EVs, 25 μl LPS-As-NSC-EVs, 50 nmol miRNA-22-3p agomir, or 25 μl LPS-As-NSC-EVs + 50 nmol miRNA-22-3P antagomir. The Basso, Beattie, and Bresnahan (BBB) open-field test (performed at days 1, 4, 7, 14, 21, and 28 post-injury) and the inclined plane test (performed at weeks 1, 2, 3, and 4 post-injury) were used to assess neurological function in two independent individuals. Animal procedures were approved by the Ethics Committee of Anhui Medical University (No. 272 20,190,164 and LLSC 20,190,154) in accordance with the guidelines of the Declaration of Helsinki revised in Edinburgh in 2000. All rats were housed in conditions of controlled temperature and humidity with a 12-h light/dark cycle.

Spinal cords were obtained from the SCI rats and incubated in 4% paraformaldehyde for 30 min. To evaluate the injury area of the lesion sites, the spinal cord was cut into 4–5-μm-thick cross-sections 4 weeks after SCI. The sections 3 mm distant from the injured center were subjected to hematoxylin–eosin staining. For immunofluorescence staining, the spinal cord was cut into a 4-μm-thick longitudinal slice (centered on the epicenter of the injured lesion) by using a Leica RM2135 electric slicer.

### Immunofluorescence staining

The fixed NSCs and a 4-μm-thick longitudinal slice of spinal cord (centered on the epicenter of the injured lesion) were prepared for immunofluorescence staining as described in our previous study [42]. The primary antibodies were used as follows: mouse anti-2′3′ cyclic nucleotide 3' phosphodiesterase (Cnpase,1:200; Abcam, UK) and rabbit anti-myelin basic protein(MBP, 1:300; Abcam, UK) for oligodendrocytes, rabbit anti-glial fibrillary acidic protein (GFAP) for astroglia (1:1000; Abcam, UK), mouse anti-nestin for NSCs (1:1000; Abcam, UK), rabbit anti-neuron-specific class III beta-tubulin (Tuj1) for neuron (1:1000; Abcam, UK),rabbit anti- SRY-Box transcription factor 10 (Sox 10) for NSCs (1:100; Abcam, UK), rabbit anti- p75 neurotrophin receptor (p 75) for NSCs (1:100, Abcam, UK), and rabbit anti-BMP2 (1:500; Affinity, USA). The secondary antibodies used were Cy3 (red, 1:50; Elabscience, China) and Alexa Fluor 488 (green, 1:50; Elabscience, 288 China). The images were observed and photographed by using a DM-6B fluorescence microscope (Leica, Germany). The percentage of positive areas was calculated using ImageJ. For cell counting, random fields containing a total of 500 cells were randomly selected. The number of positive cells was blindly quantitated in two different individuals.

### RNA extraction and quantitative PCR

Total RNA was extracted from NSCs and a 5-mm length of spinal cord tissues (center on the epicenter of the injured lesion) by using TRIzol (Gibco) according to the manufacturer’s instructions. Superscript III RT Reaction Mix (Invitrogen) was used to synthesize cDNA. RealPlex2 Mastercycler (Eppendorf) and SYBR Green master mix (Applied Biosystems) were used to perform quantitative PCR. miRNA and mRNA expression were normalized to U6 and GAPDH, respectively. The sequences of transcript-specific primers were as follows: miRNA-22-3p 5′- GCTGAGCCGCAGTAGTTCTT-3′ and 5′- GGCAGAGGGCAACAGTTCTT-3′; U6 5′- CGCTTCACGAATTTGCGTGTCAT-3′ and 5′- AACGCTTCCGAATTTGCGT-3′; KDM3A 5′-GCCAACATTGGAGACCACTTCTG -3′ and 5′-CTCGAACACCTTTGACAGCTCG-3′; Transforming growth factor β (TGF-β) 5'- CTGCTGACCCCCACTGATAC-3' and 5'- AGCCCTGTATTCCGTCTCCT-3'; GAPDH 5`-ACAACTTTGGCATTGTGGAA-3′ and 5′-GATGCAGGGATGATGTTCTG-3′.

### Western blot assay

Cells or a 3-cm length section of injured spinal cord was lysed in lysis buffer on ice. Following electrophoresis, the collected proteins were transferred to a PVDF membrane and incubated overnight with primary antibodies at 4 °C (Cnpase, 1:500, Sigma, Germany; BMP2, 1:1000, Sigma, Germany; TGF-β, 1:1000, Invitrogen, USA; p-Smad2, 1:1000, Invitrogen, USA). The membrane was then incubated with the secondary antibodies (Elabscience; 1:5000 in blocking solution) at room temperature for 1 h. The blots were then visualized using the SuperSignal West Pico enhanced chemiluminescence reagent (Thermo Scientific) and quantified using ImageJ software. The full-length original gels are included in Additional file 2: Figure S2.

### Dual-luciferase reporter analysis

To construct KDM3A-wt or KDM3A-mut, the KDM3A 3-UTR sequence containing the target gene binding site of miRNA-22-3p or a mutant reporter of KDM3A was cloned into a luciferase vector (GenScript, China). NSCs were transfected with miRNA-22-3p mimics or mimics-NC and KDM3A-wt or KDM3A-mut. Following a 48-h transfection, luciferase activity was evaluated by using the Dual-luciferase Reporter Assay System.

### Statistical analysis

Data are presented as the mean ± standard deviation. Statistical analysis was performed using SPSS software (version 16.0; Chicago, IL, USA). Student’s *t* test (two groups) or one-way analysis of variance (ANOVA) (more than two groups) with Tukey’s post hoc method was used to test the statistical significance. Statistical significance was set at *p* values < 0.05.

## Results

### LPS-As-CM promoted the differentiation of NSCs into astrocytes, leading to the failure of axonal remyelination following SCI

**Fig. 1:**
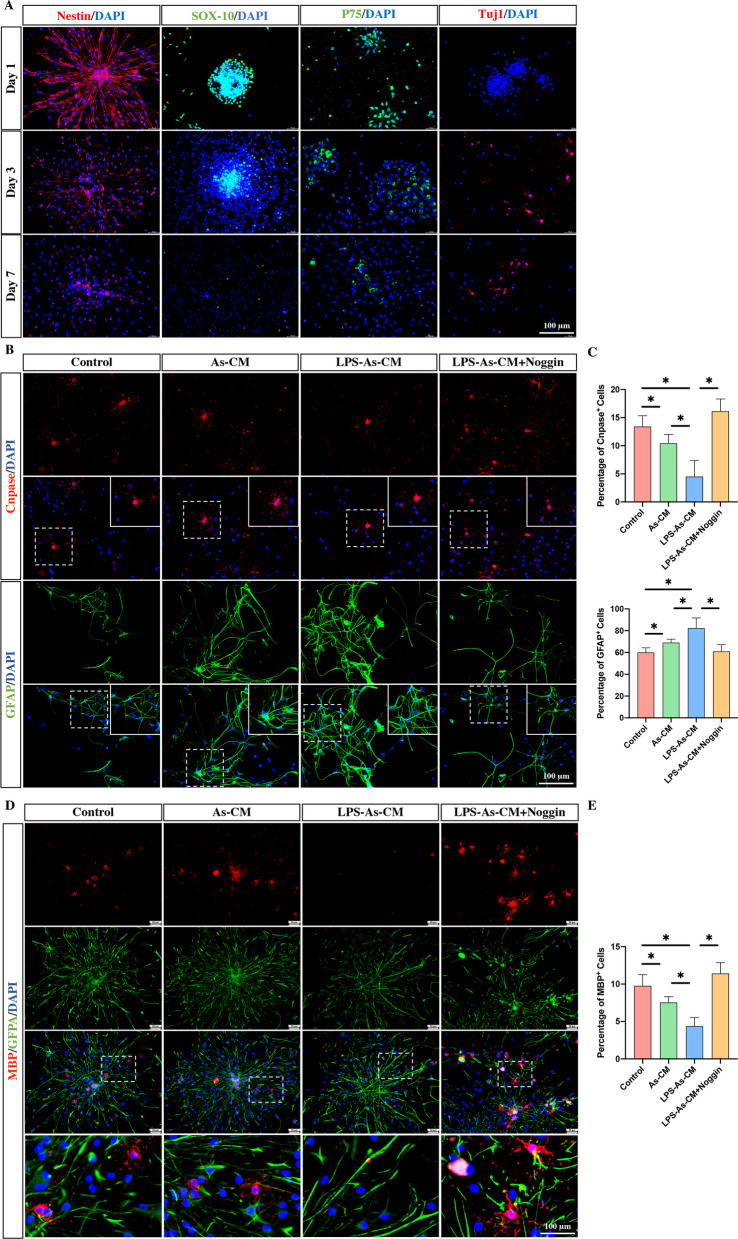
CM from astrocytes inhibited the differentiation of NSCs into oligodendrocytes. **A** Immunostaining of NSCs with markers Nestin, SOX 10, P75 and Tuj 1 at days 1,3, and 7 post-differentiation. **B** After As-CM or LPS-As-cm coculture for 7 days, the immunofluorescence results showed that the proportion of CNpase-positive oligodendrocytes was reduced and that the proportion of GFAP-positive astrocytes was increased. The LPS-As-CM-treated NSCs had a lower percentage of CNpase-positive cells and a higher percentage of oligodendrocytes than the As-CM-treated NSCs. Moreover, this LPS-As-CM-induced mediation of the differentiation of NSCs was abolished by the addition of Noggin (scale bars, 100 μm). **C** Quantitation of cell types in the presence of As-CM or LPS-As-CM with or without the addition of Noggin (*n* = 5; data are the mean ± S.D; ∗*p* < 0.05). **D** The double-immunostaining for MBP and Tuj1 showed that the percentage of MBP^+^ oligodendrocytes was reduced by the treatment of LPS-As-CM, and this LPS-As-CM-induced regulation of differentiation of NSCS was repressed by the addition of Noggin (scale bars, 100 μm). **E** Quantitation of MBP^+^ cells in the presence of As-CM or LPS-As-CM with or without the addition of Noggin (*n* = 5; data are the mean ± S.D; ∗*p* < 0.05)

To investigate whether inflammatory stimulation affects the effects of CM released from astrocytes on the differentiation of NSCs, we isolated NSCs and characterized them by immunostaining for Nestin, SOX 10, P 75 and Tuj1. The majority of NSCs were immunopositive for Nestin, Sox 10 and P 75, while NSCs seldom differentiated into the Tuj1-positive neurons at Day 1 post-differentiation (Fig. 1A). In contrast, the percentage of Nestin-, Sox 10- and P 75-positive cells was reduced, and the Tuj1-positive neurons were found at Day 3 and Day 7 (Fig. 1A). Next, we treated NSCs with CM from astrocytes or LPS-stimulated astrocytes and determined the percentage of oligodendrocytes and astrocytes after 7 days of culture by using immunofluorescence staining. Compared to the DMEM/F12-treated control groups, treatment with As-CM or LPS-As-CM increased the percentage of GFAP-positive astrocytes and reduced the proportion of Cnpase-positive oligodendrocytes (Fig. 1B, C). Moreover, LPS-As-CM-treated NSCs had a lower percentage of oligodendrocytes and a higher percentage of astrocytes than As-treated NSCs (Fig. 1B, C). Similarly, the expression of the mature oligodendroglial markers, MBP, was significantly reduced by the treatment of the LPS-As-CM, compared with the control and As-CM-treated NSCs (Fig. 1D, E). These results indicated that LPS-As-CM had a stronger effect on promoting the differentiation of NSCs toward astrocytes than As-CM-treated NSCs.

**Fig. 2:**
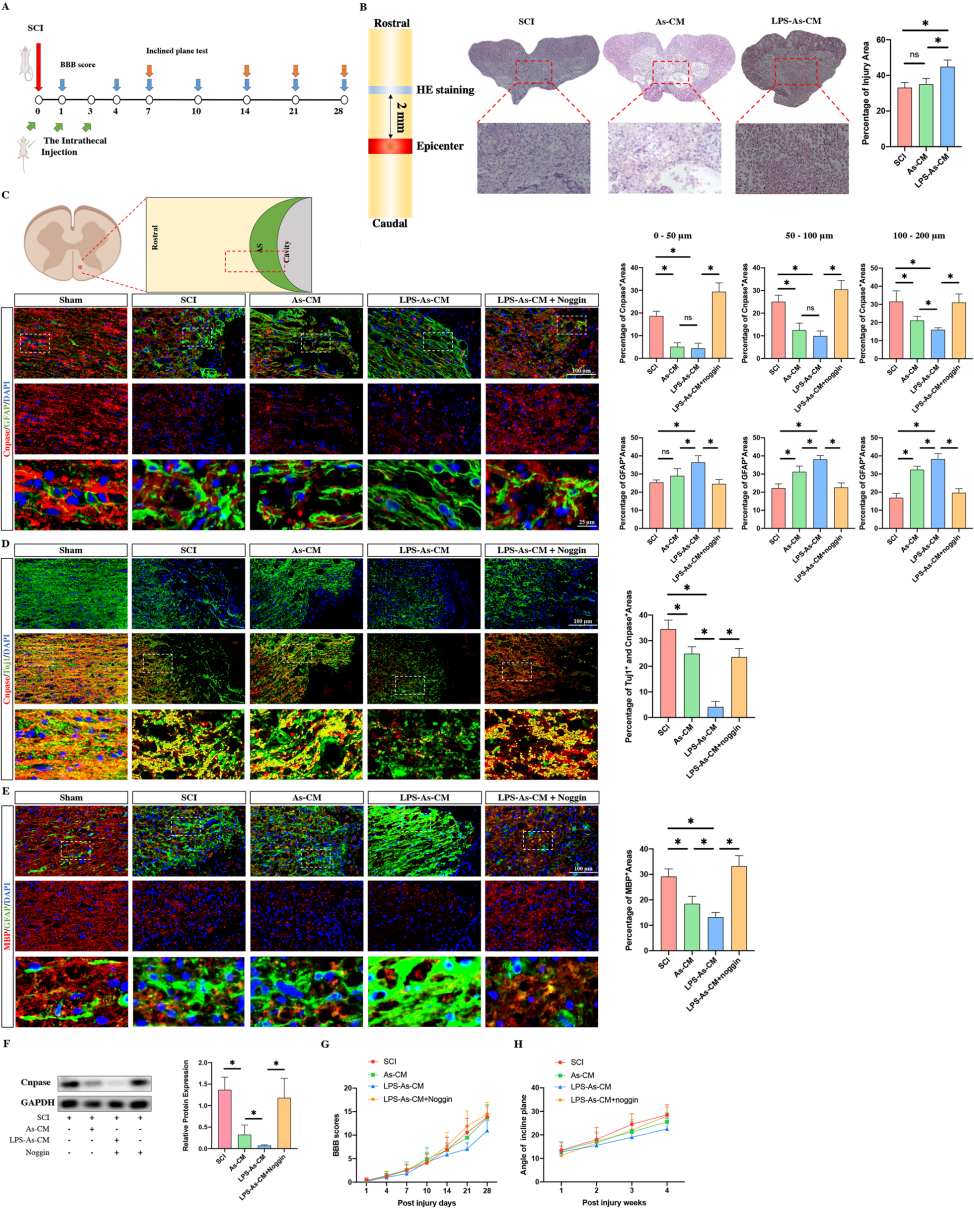
LPS-As-CM injection inhibited the regrowth of axons and worsened neurological recovery. **A** Experiment summary schematic. View of the immunofluorescently stained section in the spinal cord. The time of intrathecal injection and neurological functional assessment. **B** HE staining was performed to reveal the percentage of injured areas at 4 weeks post-injury (*n* = 5, data are the mean ± S.D; ∗*p* < 0.05, ns *p* > 0.05). **C** Area occupied by GFAP-positive scar-forming astrocytes and Cnpase-positive oligodendrocytes around the cavity at 4 weeks after SCI. The percentage of Cnpase-positive areas was reduced by the injection of As-CM and LPS-As-CM. The LPS-As-CM-induced alterations were blocked by the addition of Noggin (*n* = 5, data are the mean ± S.; ∗*p* < 0.05, ns *p* > 0.05). **D** Evaluation of the remyelination of the neurite outgrowths by assessing the double-immunostaining for Cnpase and Tuj1. The bottom panels show that the double-positive areas of Cnpase and Tuj1 (yellow areas) that were selected and used for calculation by ImageJ (*n* = 5, data are the mean ± S.D; ∗*p* < 0.05, ns *p* > 0.05). **E** The double double-immunostaining for MBP and GFAP revealed that the injection of As-CM and LPS-As-CM markedly reduced the expression of MBP within the GFAP-positive-scar boundary. The addition of Noggin blocked the LPS-As-CM-related effects in the lesion sites (*n* = 5, data are the mean ± S.; ∗*p* < 0.05, ns *p* > 0.05). **F** The western blot results showed similar data as the immunofluorescence results: 4 weeks after SCI, the expression of Cnpase was reduced by the injection of As-CM and LPS-As-CM, and the LPS-As-CM-associated downregulation of Cnpase expression was countered by the addition of Noggin (*n* = 3, data are the mean ± S.D; ∗*p* < 0.05, full length original gels are included in Additional file 2: Figure S2). **G**, **H** The BBB scores and incline plane tests in different groups post-SCI. The injection of LPS-As-CM inhibited functional recovery, and the addition of Noggin repressed the LPS-As-CM-induced effect in SCI rats (*n* = 10, data are the mean ± S. D). Significant differences in BBB scores were noted between the LPS-As-CM group and SCI group, the LPS-As-CM group and As-CM group, and the LPS-As-CM group and LPS-AS-CM + Noggin group at day 28 post-injury. Similarly, differences in the angle of the incline plane tests were found between the LPS-As-CM group and SCI group and the LPS-As-CM group and LPS-AS-CM + Noggin group at 4 weeks post-injury

A further in vivo study was carried out in which we injected As-CM or LPS-As-CM on Days 1 and 3 post-injury, respectively. After 4 weeks, the HE staining results showed that compared to the SCI or As-CM-treated SCI rats, the LPS-As-CM-injected rats had a larger injured area within the posterior white column of the injured lesions, which mainly contained the ascending tracts (Fig. 2B). Next, we cut the spinal cord into a 4-μm-thick longitudinal slice, and by using Cnpase to label myelin, we observed the axonal remyelination in the posterior white column of the lesion sites. Following SCI, a scar boundary consisting of GFAP-positive astrocytes was formed at the edge of the cavity in rats. Few Cnpase-positive cells were found in these formed astrocytic scars in the rats that received As-CM or LPS-As-CM treatment compared to the SCI rats (Fig. 2C). Moreover, the rats that received injected LPS-As-CM had the lowest percentage of Cnpase-positive area and the highest percentage of GFAP-positive areas around the edge of the cavity compared to the SCI rats and the As-CM-injected rats (Fig. 2C). In addition, we selected Tuj1 to label neurite outgrowth and measured the Cnpase and Tuj1 double-positive area to evaluate the remyelination of these neurite outgrowths in or adjacent to the glial scars. The results showed that treatment with LPS-As-CM significantly decreased the Cnpase and Tuj1 double-positive areas, compared to the SCI or As-CM-treated rats (Fig. 2D). Similarly, the expression of MBP was lowest in the rats that received the LPS-As-CM injection (Fig. 2E). Consistent with the histological results, the western blot results showed that compared to that in SCI rats, the expression of Cnpase was reduced by treatment with As-CM or LPS-As-CM, and LPS-As-CM-treated rats had the lowest expression of Cnpase (Fig. 2F). The results of the neurological functional assessments showed that compared to the SCI rats, the injection of As-CM and LPS-As-cm worsened both BBB scores (Fig. 2G) and the angle of the inclined plane test (Fig. 2H). The rats that received LPS-As-CM injection achieved the worst neurological outcome.

All these results indicated that the CM derived from LPS-stimulated astrocytes has a stronger effect in promoting the differentiation of NSCs into astrocytes, leading to the failure of axon regeneration and resulting in a worse neurological outcome.

### The LPS-As-CM-induced effects on the differentiation of NSCs were dependent on BMP2

**Fig. 3:**
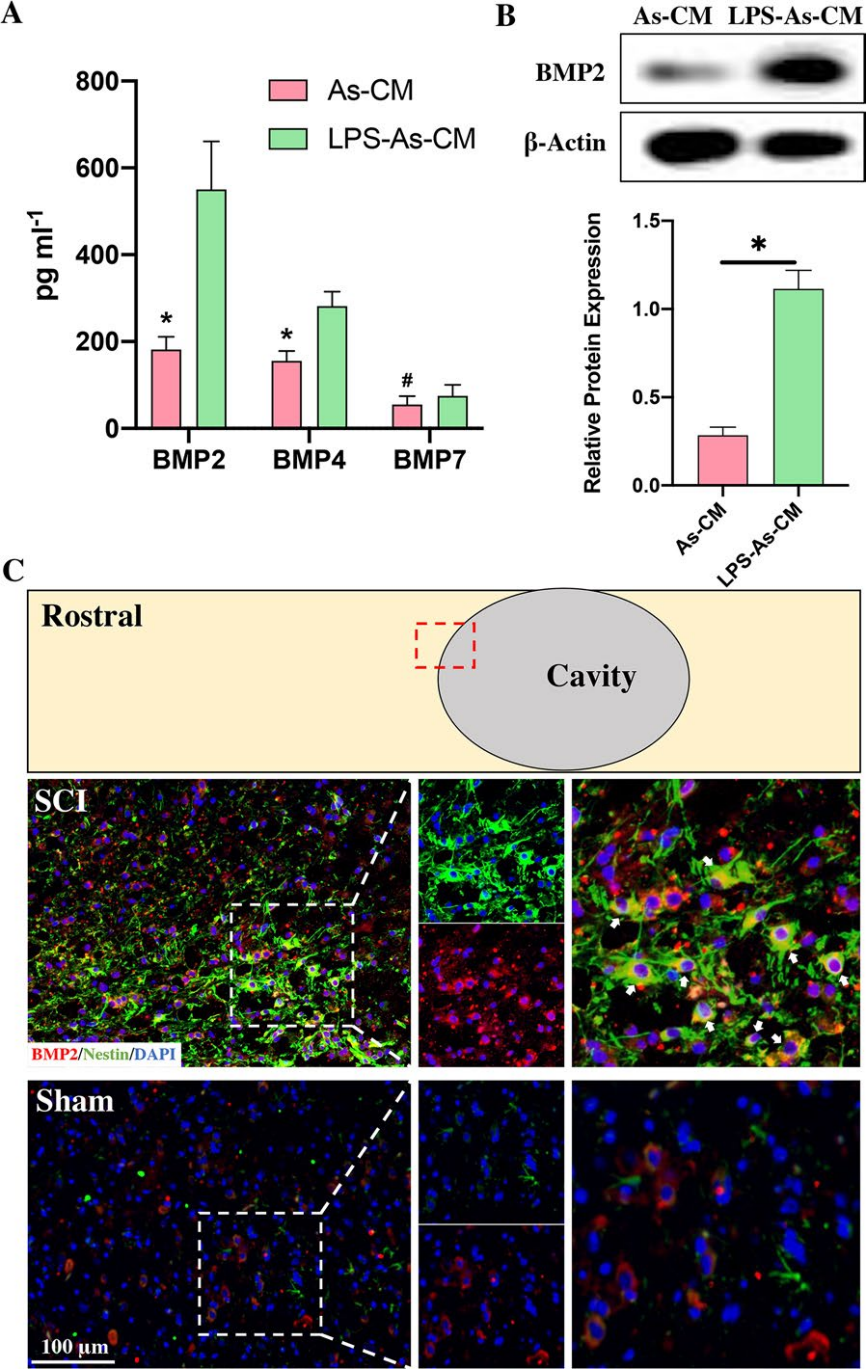
Damage to the spinal cord promoted the accumulation of Nestin-positive NSCs and increased the expression of BMP2 around the injured core. **A** The concentration of BMP2/4/7 within As-CM and LPS-As-CM was detected by ELISA. It shows that the expression of BMP2 and BMP4 in NSC-CM was upregulated by the addition of LPS-As-CM, compared with the As-CM-treated NSC-EVs (*n* = 5, data are the mean ± S.D; ∗*p* < 0.05, ns *p* > 0.05). **B** Western blot analysis showed that the expression of BMP2 in CM derived from astrocytes was increased by LPS stimulation (*n* = 3, data are the mean ± S.D; ∗*p* < 0.05, full-length original gels are included in Additional file 2: Figure S2). **C** Immunofluorescence staining of the section around the injured core at Day 3 post-SCI showed that Nestin-positive NSCs accumulated around the cavity. Compared to the sham group, the SCI rats had significantly higher expression of BMP2 in the areas around the cavity. Double staining of Nestin and BMP2 cells was observed in these areas (arrows indicate Nestin and BMP2 double-labeled cells in the areas around the cavity)

BMPs were mildly expressed in intact spinal cord but markedly expressed in oligodendrocytes, astrocytes, and microglia surrounding the damaged site post-SCI [19–22]. As astrocytes not only play a critical role in mediating inflammation, but have been reported to have a close relationship with the regrowth and remyelination of axons by regulating the differentiation of NSCs [23]. Therefore, we hypothesized that BMPs within astrocyte CM promoted the differentiation of NSCs into astrocytes. To prove this hypothesis, we first mimicked the in vivo situation in vitro by treating astrocytes with LPS to investigate whether the inflammatory stimulation of astrocytes could promote these astrocytes to release more BMPs in their conditioned medium (CM). The results showed that the expression of BMP2 and BMP 4 was increased by the treatment of LPS. In particular, BMP2 expression had the most significant increase, upregulated nearly threefold (Fig. 3A). Western blot analysis also confirmed that the LPS-treated astrocyte CM (LPS-As-CM) had a higher expression of BMP2 than that within the astrocyte CM (As-CM) (Fig. 3B). Next, we assessed the expression of BMP2 in injured lesion sites at Day 3 post-SCI. The results showed that compared with the sham rats, the SCI rats had markedly higher expression of BMP2 in the injured lesions (Fig. 3C). Moreover, in the section from these areas around the cavity, some BMP2-positive cells were also double stained with Nestin (Fig. 3C), indicating that the expression of BMP2 was increased in activated endogenous NSCs following SCI.

Next, we investigated whether these LPS-As-CM-induced effects on NSCs and SCI rats were dependent on BMP2. We added or injected Noggin (BMP antagonist) to NSCs or SCI rats in the presence of LPS-As-CM, respectively. As expected, the LPS-As-CM-induced effects on the differentiation of NSCs were significantly abolished by the addition of Noggin, resulting in a reduction in the proportion of astrocytes and an increase in the percentage of oligodendrocytes (Fig. 1B–E). Similar results were also found in vivo, which showed that the injection of Noggin together with LPS-As-CM markedly elevated the proportion of Cnpase-positive, MBP-positive, and Cnpase-Tuj1-double-positive areas in lesion sites (Fig. 2C, D, E). Similar data were also obtained by western blotting (Fig. 2F). The neurological assessments showed that the neurological outcome was recovered by the addition of Noggin in the presence of LPS-As-CM (Fig. 2G, H).

All these data suggested that inflammation following SCI could activate astrocytes to release more BMP2, which in turn promoted the differentiation of endogenous NSCs into astrocytes, which was associated with the failure of axon regeneration.

### Inflammation-stimulated As-CM affected NSC–NSC communication

**Fig. 4:**
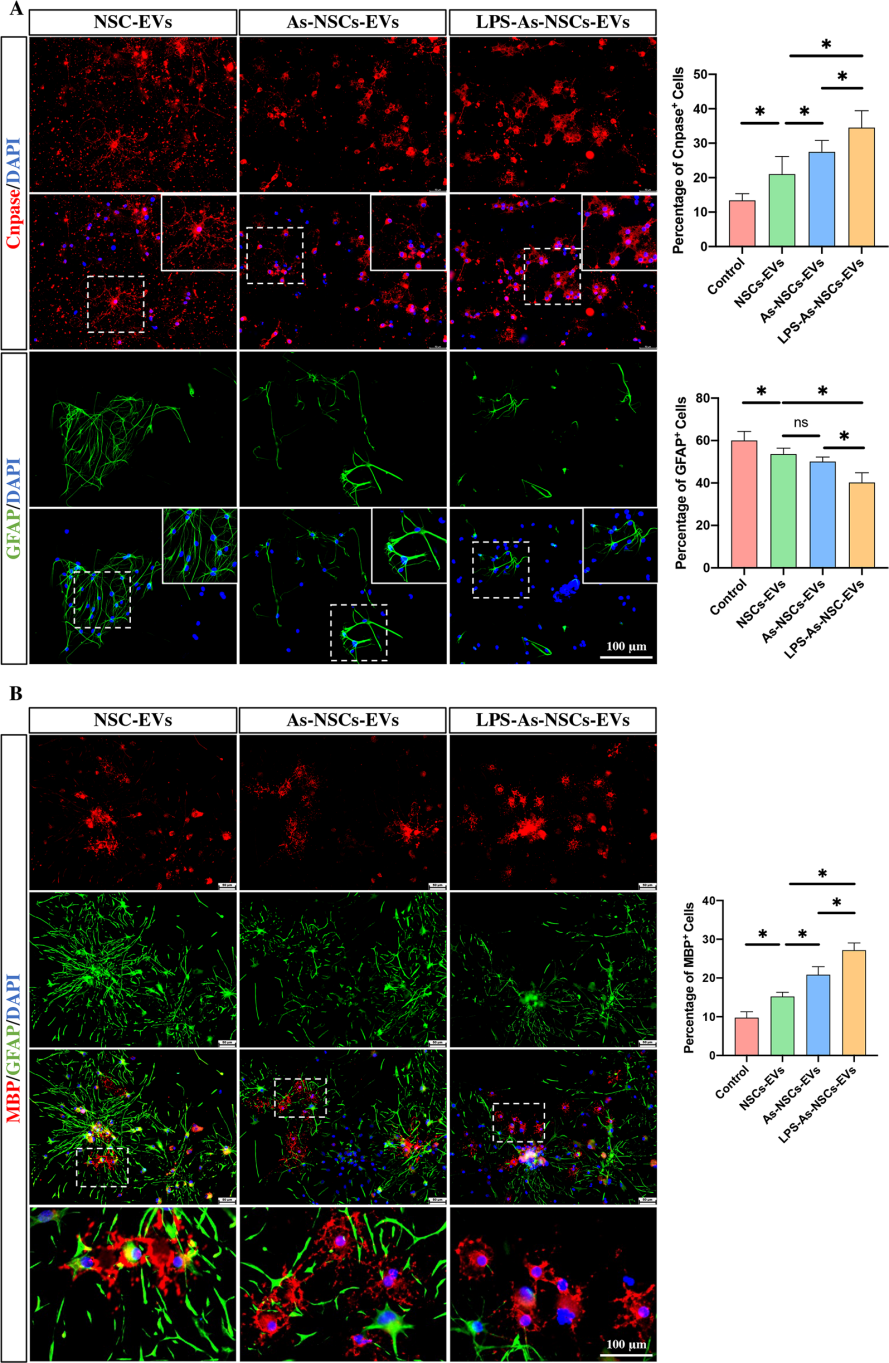
EVs derived from LPS-As-stimulated NSCs promoted the differentiation of NSCs into oligodendrocytes. **A**, **B** The immunofluorescence staining data showed that after coculturing with NSCs-EVs, As-NSCs-EVs or LPS-As-NSCs-EVs cocultured for 7 days, the NSCs treated with LPS-As-NSCs-EVs had the highest percentage of Cnpase-positive or MBP-positive oligodendrocytes and the lowest percentage of GFAP-positive astrocytes (*n* = 5; data are the mean ± S.D; *p < 0.05, ns *p* > 0.05; scale bars, 100 μm)

To investigate whether inflammation-stimulated As-CM could affect the NSCs releasing EVs and how these AS-CM stimulated NSC-EVs affected the adjacent endogenous NSCs by cell–cell communication, we first cultured NSCs for 7 days and then added 1.5 ml AS-CM or LPS-AS-CM to NSCs for 24 h, followed by switching the medium and culturing for another 24 h, and then collecting the As-CM-induced NSCs-EVs (As-NSCs-EVs) or LPS-As-CM-induced NSCs-EVs (LPS-As-NSCs-EVs). To test whether these stimulated NSCs-EVs could mediate the differentiation of NSCs, we added As-NSCs-EVs or LPS-As-NSCs-EVs to neurospheres in differentiation medium for 7 days and determined the percentage of oligodendrocytes and astrocytes by using immunofluorescence staining. The results showed that compared to the control groups, the NSCs-EVs, As-NSCs-EVs or LPS-As-NSCs-EVs increased the percentage of oligodendrocytes (Fig. 4A, B). Among all three groups, LPS-As-NSC-EV-treated NSCs had the highest proportion of oligodendrocytes and the lowest proportion of astrocytes (Fig. 4A, B), indicating that the LPS-As-NSCs-EVs had a stronger effect on promoting the differentiation of NSCs into oligodendrocytes than NSCs-EVs and As-NSCs-EVs.

**Fig. 5:**
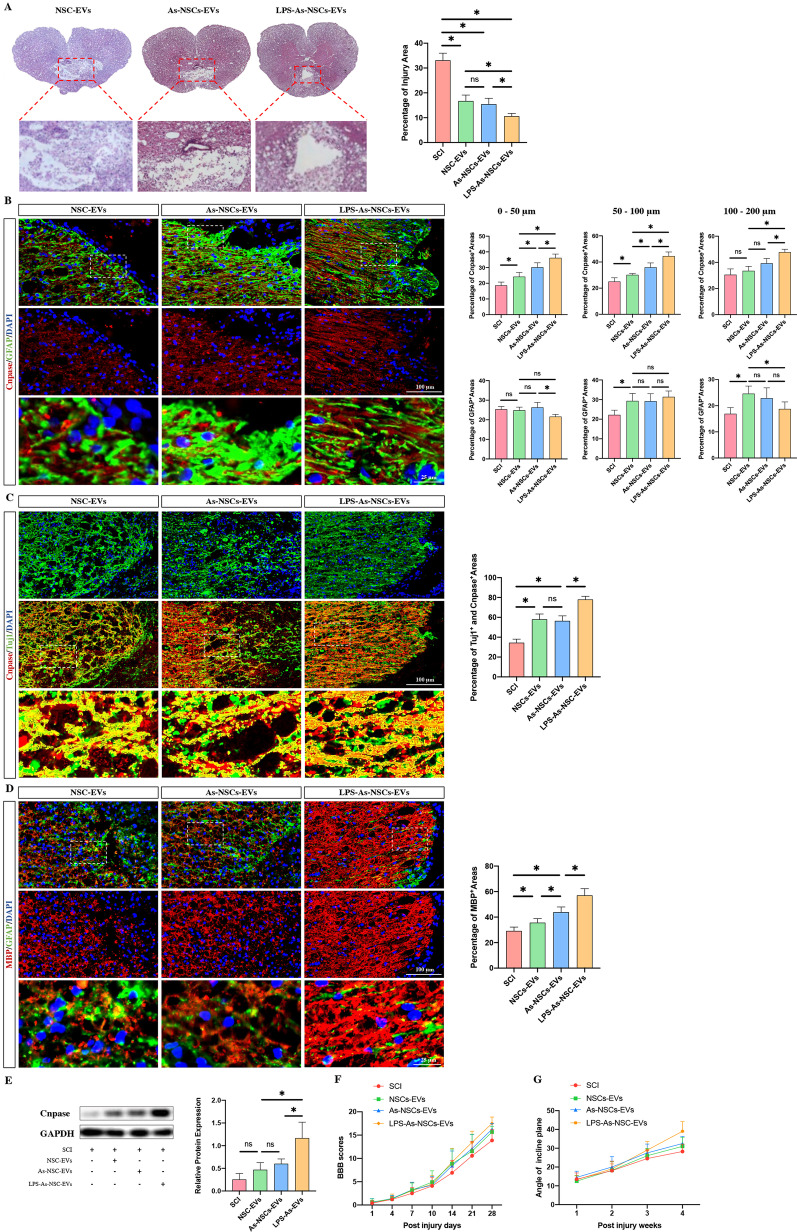
EVs derived from LPS-As-stimulated NSCs promoted axonal regeneration and remyelination, and the neurological recovery following SCI. **A** HE staining results showed that the injured areas were reduced by the injection of NSCs-EVs, As-NSCs-EVs or LPS-As-NSCs-EVs, compared to the rats that did not receive EVs injection. The LPS-As-NSC-EVs-injected rats had the smallest injured areas (*n* = 5, data are the mean ± S.D; ∗*p* < 0.05, ns *p* > 0.05). **B**–**D** The LPS-As-NSCs-EVs had the strongest effects in promoting the remyelination around the cavity at week 4 post injury (*n* = 5, data are the mean ± S.D; ∗*p* < 0.05, ns *p* > 0.05). **E** The western blot results revealed the alteration in Cnpase expression in the injured core at week 4 post-injury (*n* = 3, data are the mean ± S.D; ∗*p* < 0.05, full length original gels are included in Additional file 2: Figure S2). **F**, **G** Consistent with histological data, the LPS-As-NSCs-EVs-treated rats showed a better neurological recovery compared to the NSCs-EVs or As-NSCs-EVs-treated rats (*n* = 10, data are mean ± S. D). Significant differences in BBB scores were noted among all 3 groups at day 28 post-SCI. Similarly, differences in the angle of incline plane tests were found between the SCI and LPS-As-NSCs-EVs, As-NSCs-EVs, and LPS-As-NSCs-EVs groups at week 4 post-injury

In vivo, SCI rats received NSC-EV, As-NSC-EV, or LPS-As-NSC-EV injections at the time of injury and on days 1 and 3 post-injury. HE staining results showed that the injection of these NSC-EVs reduced the injured areas compared to SCI rats. Moreover, the LPS-As-NSC-EV-treated rats had the smallest injured area compared to the NSC-EV or the As-CM-NSC-EV-treated rats (Fig. 5A). The immunofluorescence staining results of 4-week tissue showed that all the injected rats had higher oligodendrocyte-positive areas surrounding cavities (Fig. 5B, C, D). Consistent with the in vitro results, the LPS-As-NSC-EV-injected rats had the highest oligodendrocyte expression and Cnpase-Tuj1 double-positive areas (Fig. 5C). Western blot analysis showed similar results (Fig. 5E). As expected, the injection of LPS-As-NSCs-EVs resulted in better BBB scores and a higher angle of incline plane compared to the other groups (Fig. 5F, G).

**Fig. 6:**
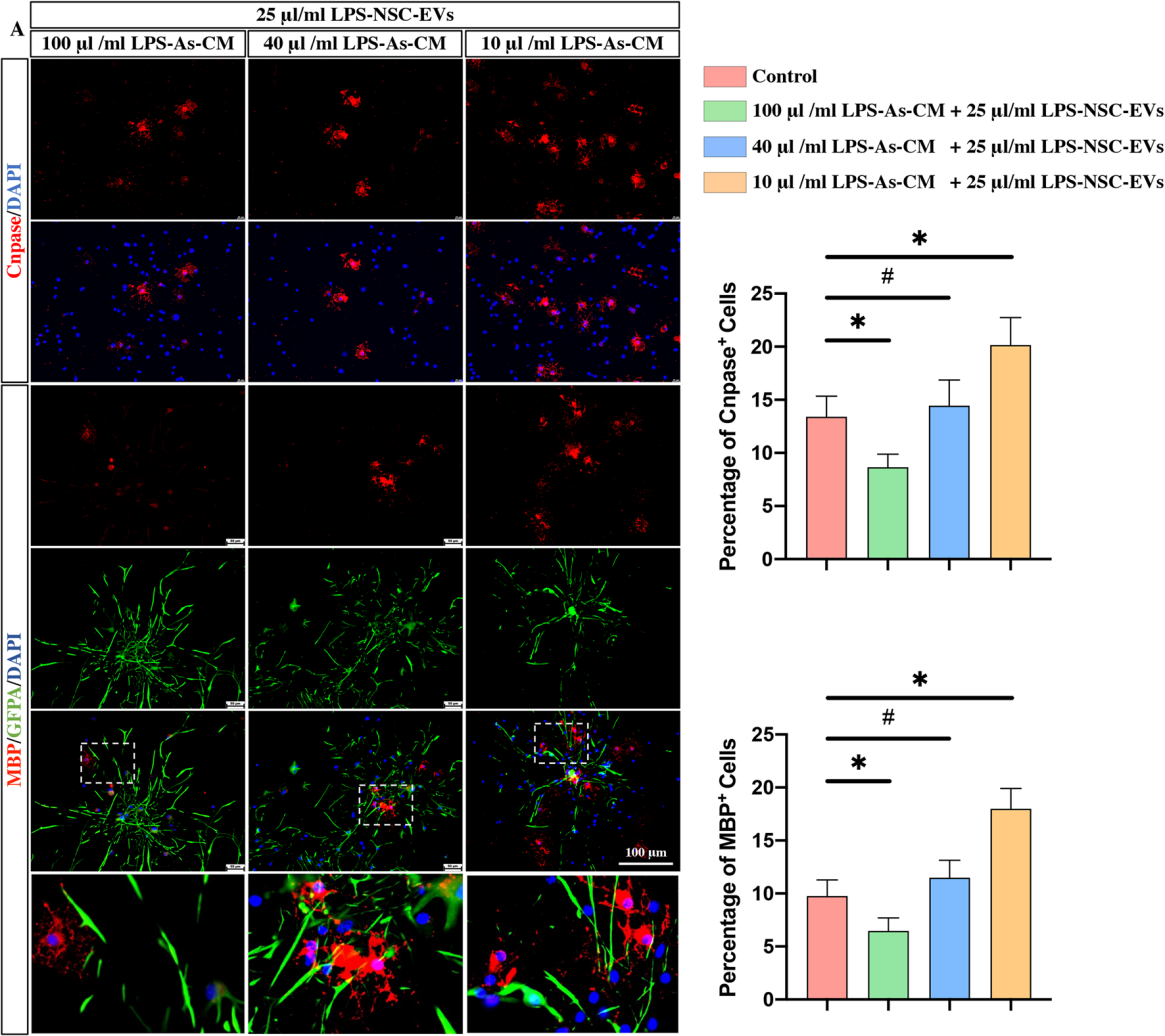
LPS-NSC-EVs act against LPS-As-CM leading the differentiation of NSCs into oligodendrocytes. The NSCs were cultured with different concentrations of LPS-As-CM and LPS-As-NSC-EVs, immunofluorescence staining data revealed that with the reduction of the dose of the LPS-As-CM, the differentiation of NSCs switched from astrocytes to oligodendrocytes (*n* = 5, data are the mean ± S.D; ∗*p* < 0.05, ns *p* > 0.05)

All these data indicated that the EVs derived from LPS-As-CM-stimulated NSCs had a stronger effect on promoting the regeneration of oligodendrocytes, compared with the none-stimulated NSC-EVs and the As-CM-stimulated NSC-EVs. Therefore, we hypothesized that the endogenous EVs released from NSCs, which received the signal from inflammation-stimulated astrocytes, might work as a feedback regulator to against the astrocyte-released cytokines and inhibit the over-formation of glial scar. To prove this hypothesis, we culture NSCs with different concentration of LPS-As-CM and LPS-As-NSC-EVs. It showed that with the reduction of the dose of the LPS-As-CM, the differentiation of NSCs switched from astrocytes to oligodendrocytes (Fig. 6).

### LPS-As secreted BMP2 to upregulate the expression of miRNA-22-3p in NSCs-EVs

Micro-RNAs (miRNAs) are short RNA molecules of 20–22 nucleotides in length that work as negative regulators by degrading or repressing the targeted mRNAs in a variety of cellular processes [44]. The biological effects of EVs on regulating physiological or pathophysiological functions are closely associated with these miRNAs [44, 45]. To investigate whether the expression miRNAs within NSC-EVs could be regulated by the addition of LPS-As-CM, we detected the expression of axonal regeneration-related and remyelination-related miRNAs within the EVs from the As-CM-treated NSCs or the LPS-As-CM-treated NSCs. The data revealed that miR-22-3p had the most significantly increase among these miRNAs and upregulated more than eightfold (Additional file 3: Fig. 2A). Combined with previous studies that miRNA-22 was able to mediate both neural regrowth and inflammatory processes following neurotrauma [46–48]. Therefore, we hypothesized that by releasing BMP2, LPS-As-CM might regulate the expression of miRNA-22 in NSCs-EVs, which in turn acted as a feedback loop to inhibit the differentiation of NSCs into astrocytes.

**Fig. 7:**
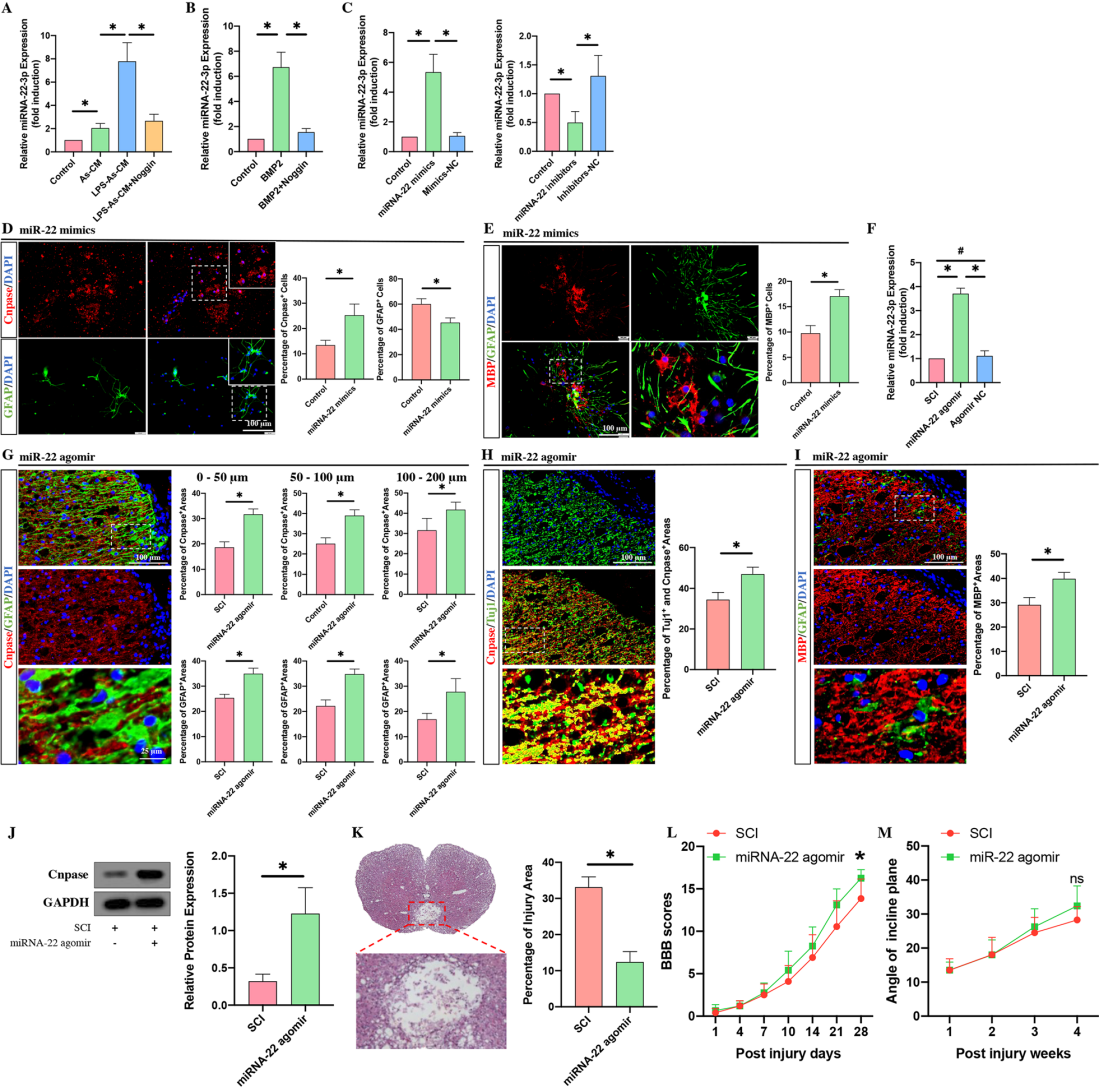
miRNA-22-3p had a biological effect similar to that of NSCs-EVs in regulating the differentiation of NSCs in vitro and promoting axonal regrowth and remyelination in vivo. **A**, **B** The PCR results showed that the expression of miRNA-22-3p in NSCs-EVs was increased after coculture with LPS-AS-CM or BMP2 for 24 h (**A**) and was inhibited by the addition of Noggin in the presence of LPS-AS-CM or BMP2 (**B**) (*n* = 3, data are the mean ± S. D., ∗*p* < 0.05). **C** The effects of the miRNA-22-3p mimics and inhibitors were confirmed by PCR after 24 h of transfection (*n* = 3, data are the mean ± S. D., ∗*p* < 0.05). **D**, **E** The transfection of miRNA-22-3p promoted the differentiation of NSCs into oligodendrocytes (*n* = 5; data are the mean ± S.; *p < 0.05). **F** The effect of miRNA-22-3p agomir in SCI rats was confirmed by PCR after continuous injection for 3 days (*n* = 3, data are the mean ± S. D., ∗*p* < 0.05). **G**,**H**,**I** The injection of miRNA-22-3p agomir increased the expression of Cnpase and MBP in the astrocytic scar and the percentage of Cnpase- and TUJ1- double-positive areas in lesion sites at week 4 post-SCI (*n* = 5; data are the mean ± S.D.; *p < 0.05). **J** The western blot results showed that the expression of Cnpase 4 weeks after SCI was increased by the injection of miRNA-22-3p agomir (*n* = 3, data are the mean ± S. D., ∗*p* < 0.05). **K** HE staining results showed that the percentage of the injured area was reduced by the injection of miRNA-22 agomir (*n* = 5, data are the mean ± S.D.; ∗*p* < 0.05, ns *p* > 0.05). **L**, **M** miRNA-22 agomir promoted the recovery of neurological function following SCI (*n* = 10, data are the mean ± S.D.; ∗*p* < 0.05, ns *p* > 0.05)

To prove this hypothesis, we first cocultured NSCs with As-CM or LPS-As-CM for 24 h, switched the medium to DMEM/F12 for another 24 h, collected the EVs and determined the expression of miRNA-22-3p by PCR. The results showed that the LPS-As-CM-treated NSCs had the highest expression of miRNA-22-3p compared to the control and As-CM-treated NSCs (Fig. 7A). To determine whether this LPS-As-CM-induced upregulation of miRNA-22-3p in NSCs-EVs was associated with BMP2, we added BMP2 to NSCs with or without Noggin for 24 h, followed by switching the medium and assessing miRNA-22-3p in EVs. BMP2 treatment markedly increased the expression of miRNA-22-3p in EVs compared to that in the control groups, and this BMP2-induced miRNA-22-3p increase was abolished by the addition of Noggin (Fig. 7B). Then, we added Noggin to NSCs for 24 h in the presence of LPS-As-CM, and as expected, the expression of miRNA-22-3p was reduced in NSCS-EVs (Fig. 7A). All these data suggested that LPS-As-CM was able to increase the expression of miRNA-22-3p in NSCs-EVs by secreting BMP2.

### LPS-As-NSCs-EVs promoted the differentiation of NSCs into oligodendrocytes partly through miRNA-22-3p

**Fig. 8:**
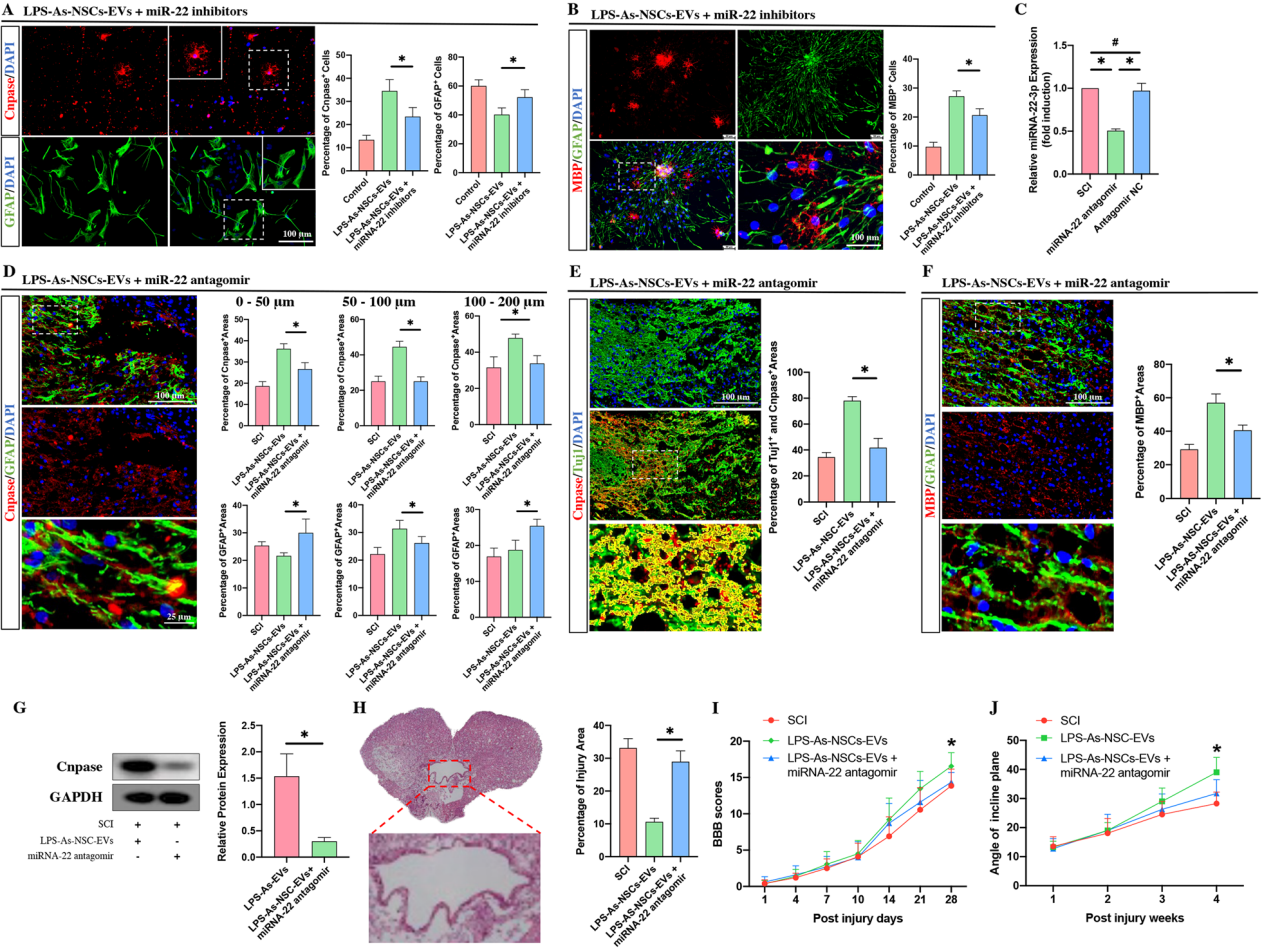
miRNA-22-3p within NSCs-EVs contributed to the LPS-As-NSCs-EVs-induced effects in the regulation of NSCS differentiation and promotion of axon regrowth. **A**, **B** The transfection of miRNA-22-3p inhibitors reduced the proportion of Cnpase-positive and MBP-positive- oligodendrocytes and increased the proportion of GFAP-positive astrocytes in the presence of LPS-As-NSCs-EVs (*n* = 5; data are the mean ± S.D.; *p < 0.05). **C**The expression of miRNA-22-3p was reduced by a 3-day continuous injection of antagomir (*n* = 3; data are the mean ± S.D.; *p < 0.05, ns *p* > 0.05). **D**–**F** The injection of miRNA-22-3p antagomir repressed the LPS-As-NSC-EVs-induced effects on promoting the remyelination following SCI. (*n* = 5; data are the mean ± S.D.; *p < 0.05). **G** Western blot results confirmed that the expression of Cnpase was decreased by the injection of miRNA-22-3p antagomir at week 4 post-injury (*n* = 3; data are the mean ± S.D.; *p < 0.05). **H** The injection of miRNA-22 antagomir increased the percentage of injured areas at 4 weeks post-injury (*n* = 5, data are the mean ± S.D.; ∗*p* < 0.05, ns *p* > 0.05). **I**, **J** LPS-As-NSCs-EVs-induced neurological recovery was inhibited by the injection of miRNA-22-3p (*n* = 10, data are the mean ± S. D.; ∗*p* < 0.05 between the LPS-As-NSCs-EVs group and the LPS-As-NSCs-EVs + miRNA-22-3p antagomir group)

To prove whether the biological effects of LPS-As-NSCs-EVs were associated with miRNA-22-3p, we investigated the effects of miRNA-22-3p on the differentiation of NSCs. The neurospheres were transfected with miRNA-22-3p mimics (Fig. 7C) and adherently cultured for 7 days with differentiation medium, and then, immunofluorescence staining was used to determine the percentage of oligodendrocytes and astrocytes. As expected, the percentage of oligodendrocytes was increased with the reduction in the proportion of astrocytes after miRNA-22-3p mimics transfection (Fig. 7D, E). To further prove the relationship between the LPS-As-NSCs-EVs and miRNA-22-3p, the neurospheres were transfected with miRNA-22-3p inhibitors (Fig. 7C) and then cultured for 7 days in the presence of LPS-As-NSCs-EVs. Compared to the LPS-As-NSCs-EVs treated NSCs, the percentage of oligodendrocytes was reduced by the transfection of miRNA-22-3p inhibitors (Fig. 8A, B), indicating that the LPS-As-NSC-EV-induced effects on the differentiation of NSCs were partly associated with miRNA-22.

In vivo, the miRNA-22-3p agomir/antagomir was used to up/downregulate miRNA-22-3p in injured lesions. Consistent with the in vitro results, the injection of miRNA-22-3p agomir upregulated the expression of miRNA-22 at day 3 post-injury (Fig. 7F), markedly promoted the regeneration of oligodendrocytes in the glial scar boundary around the cavity (Fig. 7G–J), reduced the injured area in lesion sites (Fig. 7K), and improved the neurological functions (Fig. 7L, M). In contrast, the downregulation of miRNA-22 in NSCs significantly decreased the percentage of oligodendrocytes (Fig. 8A, B). In vivo, the miRNA-22 expression was reduced by the injection of miRNA-22 antagomir (Fig. 8C), resulting in a reduction in oligodendrocytes around the cavity (Fig. 8D–G), an increase in the percentage of injured areas (Fig. 8H), and worse neurological outcomes (Fig. 8I, J).

### LPS-As-NSCs-EVs mediated the differentiation of NSCs through the miRNA-22/KMD3A/TGF-β axis

**Fig. 9:**
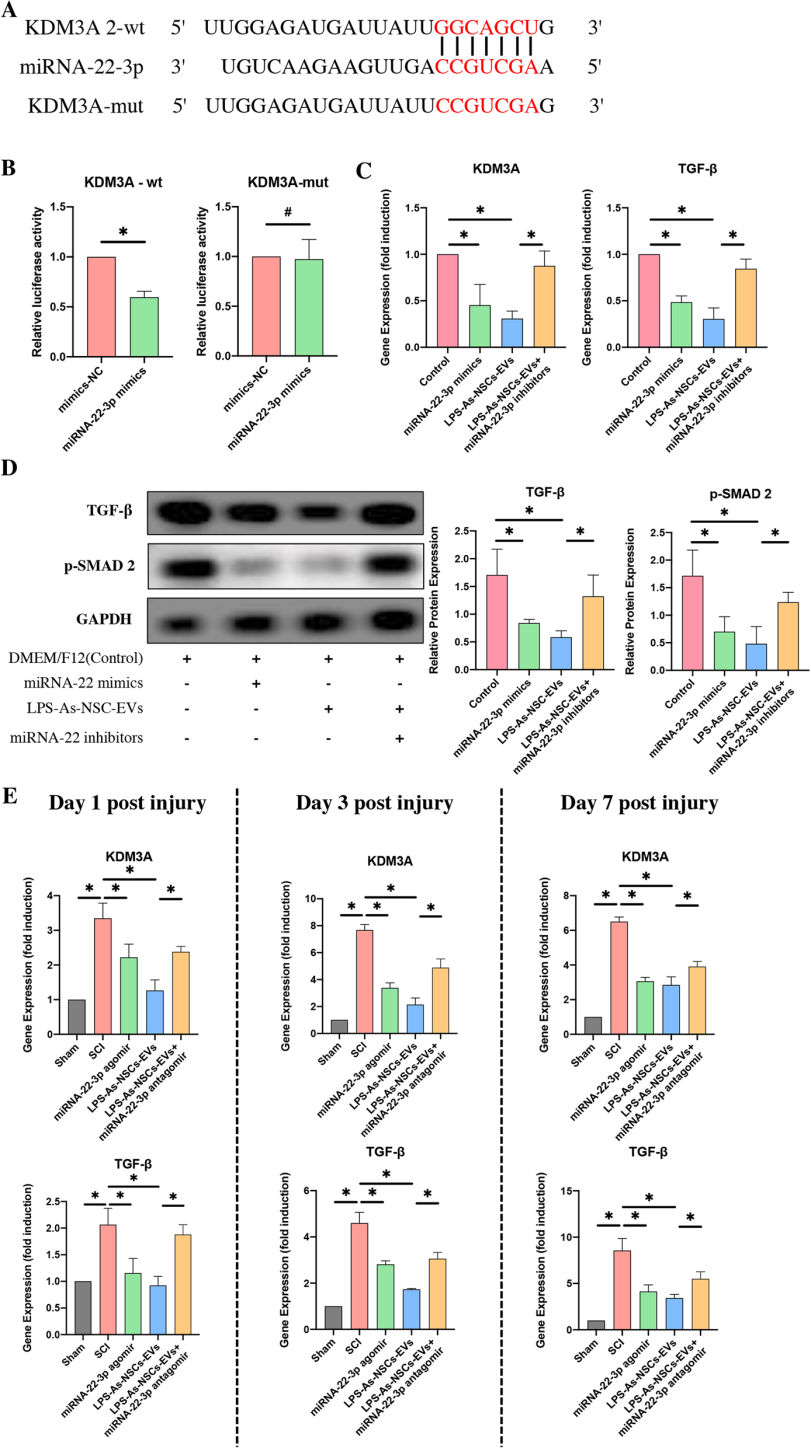
miRNA-22 inhibited the TGF-β signaling by targeting KDM3A. **A** The target sequence for miRNA-22-3p in the 3′-UTR of KDM3A and the mutated target sequence. **B** KDM3A-wt or KDM3A-mut was transfected into NSCs together with miRNA-22-3p mimics or mimics-NC. Dual-luciferase reporter analysis confirmed the direct recognition of the KDM3A 3′-UTR by miRNA-22-3p (*n* = 3; data are the mean ± S.D.; ∗*p* < 0.05, ns *p* > 0.05). **C** The transfection of miRNA-22-3p mimics and the coculture of LPS-As-NSCs-EVs into NSCs downregulated the expression of KDM3A and TGF-β after transfection or culture for 24 h. In contrast, the transfection of miRNA-22-3p inhibitors into NSCs upregulated the expression of these 2 genes in the presence of LPS-As-NSCs-EVs (*n* = 3; data are the mean ± S.D.; ∗*p* < 0.05). **D** Western blot analysis revealed that the expression of TGF-β and p-Smad 2 was downregulated by the transfection with miRNA-22-3p mimics or the coculture with LPS-As-NSCs-EVs for 24 h. Moreover, the LPS-As-NSCs-EVs-induced downregulation of TGF-β and p-Smad 2 expression was countered by the transfection with miRNA-22-3p inhibitors (*n* = 3; data are the mean ± S.D.; ∗*p* < 0.05). **E** Consistent with the in vitro data, the injection of miRNA-22-3p agomir or LPS-As-NSCs-EVs reduced the expression of TGF-β and p-Smad 2 at different time points following SCI. Moreover, this LPS-As-NSCs-EVs-induced mediation of the expression of TGF-β and p-Smad 2 was partly abolished by injection of the miRNA-22-3p antagomir (*n* = 3; data are the mean ± S.D.; ∗*p* < 0.05)

To further investigate the mechanism by which miRNA-22 regulates the differentiation of NSCs, the TargetScan database was used to predict the downstream molecular genes of miRNA-22-3p. Among the top 50 database-predicted targeted genes, 8 genes (HOMER1, FRAT2, GRM5, TGFBR1, KDM3A, RGS2, ARRB1, and YWHAZ) were reported to be associated with axonal/neuronal regeneration/or neurological diseases. Then, we detected the expression of these 8 genes in NSCs after the transfection of miR-22 mimics by PCR. The results showed that the expressions of HOMER1, TGFBR1 and KDM3A were decreased (Fig. 9C; Additional file 3: Figure S3B). Next, the dual-luciferase reporter assay was performed to identify the association between miRNA-22 and these 3 genes. The results revealed that the luciferase activity was not altered by the transfection of miRNA-22 mimics in the HOMER1 and TGFBR1 groups (Additional file 3: Figure S3C, D). In KDM3A groups, the luciferase activity was markedly reduced by the transfection of miRNA-22 mimics in the 3′UTR-WT groups. In contrast, the alteration of luciferase activity was not noted in the 3′UTR-mut groups (Fig. 9B).

KDM3A, which has been reported to be associated with fibrosis by upregulation of TGF-β [49], was bound by miRNA-22-3p at position 28–34 (Fig. 9A). In SCI, TGF-β has been proven to promote not only the formation of glial or fibrotic scars in injured lesions but also the differentiation of NSCs into astrocytes via its downstream protein Smad 2/3 [43, 50]. Therefore, we hypothesized that miRNA-22 could regulate the differentiation of NSCs by targeting KMD3A and repressing the TGF-β signaling pathway. To prove this hypothesis, we investigated the expression of the KDM3A and its downstream gene TGF-β in NSCs after transfection with miRNA-22 mimics by PCR. As expected, the expression of both KDM3A and TGF-β was reduced by transfection with miRNA-22 mimics (Fig. 9C). The results of western blot analysis were consistent with the PCR data. The transfection of miRNA-22 mimics reduced the expression of TGF-β and p-SMAD2 in NSCs (Fig. 9D). Similarly, in vivo, the injection of miRNA-22 agomir reduced the expression of KDM3A and TGF-β in injured lesions at different time points following SCI (Fig. 9E). All these data indicated that miRNA-22 was able to repress TGF-β signaling by targeting KDM3A.

To further investigate whether LPS-As-NSCs-EVs were able to downregulate TGF-β signaling via the miRNA-22/KDM3A axis, we determined the expression of KDM3A and TGF-β in NSCs after 24 h of coculture with LPS-As-NSCs-EVs, revealing that the addition of LPS-As-NSCs-EVs significantly decreased the expression of these genes (Fig. 9C). Moreover, this LPS-As-NSCs-EVs-induced downregulation of the expression of these 2 genes in NSCs was abolished by the transfection of miRNA-22 inhibitors (Fig. 9C). Consistent with these data, western blot analysis showed that the expression of TGF-β and p-SMAD2 in NSCs was reduced by the culture of LPS-As-NSC-EVs, and that this reduction was abolished by the transfection of miRNA-22 inhibitors (Fig. 9D). In vivo, the injection of LPS-As-NSCs-EVs markedly reduced the expression of KMD3A and TGF-β at different time points following SCI, and this LPS-As-NSCs-EVs-induced effect was countered by the coinjection of miRNA-22 antagomir (Fig. 9E). This result suggested that LPS-As-NSCs-EVs downregulated KDM3A/TGF-β expression partly through miRNA-22.

## Discussion

Astrocytes are found throughout the CNS, with each cell containing numerous stellate processes forming a well-delineated bushy territory, which forms intimate contacts with synapses and other neural cells [51]. In the acute phase of SCI, astrocytes rapidly form astrocytic barriers surrounding the injured lesion core and separate the inflammatory cells from adjacent tissues. Thus, astrocytic scars limit destructive inflammation and protect surviving nerve cells [52, 53]. However, the overformation of astrocytes also acts as a barrier, which is considered to be one of the causes for the failure of axon regeneration [54, 55]. In addition, recent studies have suggested that astrocytes are immune cells that propagate inflammation, which plays a critical role in the process of neurotrauma, including SCI [4, 51, 56]. As chemokines and cytokines immigrate and accumulate in injured lesion sites following SCI, they act as triggers to activate astrocytes. These activated astrocytes release various proinflammatory cytokines, which are helpful in the early period of injury for accelerating debris clearance. However, this also causes destructive inflammatory damage to the spared adjacent neural tissues [57, 58]. In addition, by producing cytokines, astrocytes are able to communicate with adjacent cells and are likely to participate in the de/remyelination of axons, neurite outgrowth, and formation of glial scars [59–63]. Therefore, we considered astrocytes to act as a “bridge” to connect early inflammation following SCI and nerve cell regeneration. They are stimulated by inflammation and transmits inflammatory signals to NSCs to mediate the differentiation of these NSCs.

In the present study, we found that by releasing cytokines, inflammatory stimulated astrocytes inhibited the differentiation of endogenous NSCs into oligodendrocytes, resulting in the failure of axonal regrowth and remyelination in the astrocytic scars surrounding the cavity. Further study showed that LPS stimulation markedly increased the release of BMP2 by astrocytes and that the LPS-As-CM-induced effects on NSCs or SCI rats were repressed by the addition of Noggin, suggesting that these LPS-As-CM-induced effects were associated with BMP2. Taken together, inflammation triggered astrocytes to release proinflammatory cytokines, accelerating debris clearance. On the other hand, astrocytes released BMP2 to promote the differentiation of endogenous NSCs into astrocytes, forming a glial scar boundary, which in turn limited the spread of destructive inflammation. However, this feedback also led to the inhibition of axon regeneration and the failure of remyelination.

In the present study, we also studied the alteration of NSCs in response to cytokines from inflammatory stimulated astrocytes. EVs are likely messengers that directly deliver molecular information between cells, inducing multiple biological functions [64]. The proinflammatory environment following SCI can regulate the specific RNA and proteins in NSC-derived EVs, which induce various responses in adjacent targeted cells and regulate their biological functions [65]. A study from Morton et al. found that EVs derived from NSCs were able to target microglia and induce regulation of the physiology and morphology of these microglia [66]. In some studies, NSC-derived EVs have been directly used to treated neurotrauma, which achieved promising outcomes by influencing the inflammation, apoptosis, and regeneration of neurons and axons [35, 36, 67, 68]. Herein, in the present study, we found that NSCs-EVs were able to promote neurological recovery following SCI. Moreover, NSCs-EVs stimulated by LPS-As-CM had more significant effects on the remyelination of axons and led to better neurological outcomes in SCI rats than NSCs-EVs and As-CM-NSCs-EVs. This result suggested that in response to the cytokines released from the astrocytes, the endogenous NSCs had cell–cell communication with the adjacent NSCs and promoted the differentiation of these NSCs into oligodendrocytes, promoting the regrowth and remyelination of axons.

Previous studies have revealed that miRNAs within EVs play a critical role in the effects exerted by EVs [27, 29]. In the present study, we found that the LPS stimulation significantly increased the expression of BMPs expression within astrocyte-CM. Comparted with BMP4 and BMP 7, BMP2 in CM had the most significant increase after the stimulation of LPS; the expression of miR-22 within NSC-EVs was significantly increased by the addition of BMP2; the addition of Noggin to NSCs could directly abolish the BMP2- or LPS-AS-CM-induced effects on the upregulation of miR-22 within NSC-EVs. It indicated that BMP2 derived from astrocytes had a closely relationship with the upregulation of miR-22 expression within NSC-EVs. In addition, several lines of evidence indicated that the enhancement effect of LPS-As-CM-NSC-EVs on the mediation of NSC differentiation might be associated with miR-22. First, compared to As-CM, LPS-As-CM had a higher level of BMP2 expression. In terms of effect on NSCs, LPS-As-CM had a stronger effect on the upregulation of miR-22 within EVs than As-CM. Furthermore, this enhanced effect of LPS-As-CM on the upregulation of miR-22 was countered by the addition of Noggin. Second, the miR-22 mimics/agomir had a similar effect as NSC-EVs. In vitro, both miR-22 mimics and NSC-EVs were able to promote the differentiation of NSCs into oligodendrocytes. In vitro, both of them increased the regeneration of axons in injured lesions. The LPS-As-CM-stimulated NCS-EVs, which had a higher expression of miR-22, showed a stronger effect on the regulation of NSC differentiation and the regeneration of axons following SCI. Moreover, this effect was abolished by the addition of miR-22 inhibitor/antagomir.

The present study of the mechanisms of miRNA-22 showed that miRNA-22 was able to inhibit TGF-β signaling by targeting KDM3A. TGF-β is a versatile cytokine that is upregulated following SCI. TGF-β is not only associated with inflammation but also has a strong effect on the formation of glial scars. It can be activated by inflammation and induce the reactivity and generation of astrocytes around the injured cores. Moreover, TGF-β has also been shown to promote astrocytes to produce more chondroitin sulfate proteoglycans (CSPGs), which, in turn, inhibit the outgrowth of axons [3, 69, 70]. Therefore, we hypothesized that the upregulation of miRNA-22 following SCI could directly repressed TGF-β signaling, by which miRNA-22 promoted the differentiation of endogenous NSCs into oligodendrocytes, resulting in longer-distance regrowth of remyelinated axons following SCI. In addition, miRNA-22 was also reported to alleviate cerebral ischemic injury by repressing BMP2 signaling by targeting KDM6B [71]. Taken together, these results suggest that miRNA-22 acts as a negative regulator of TGF-β and BMP2 signaling to prevent the overformation of astrocytic scars following SCI.

**Fig. 10:**
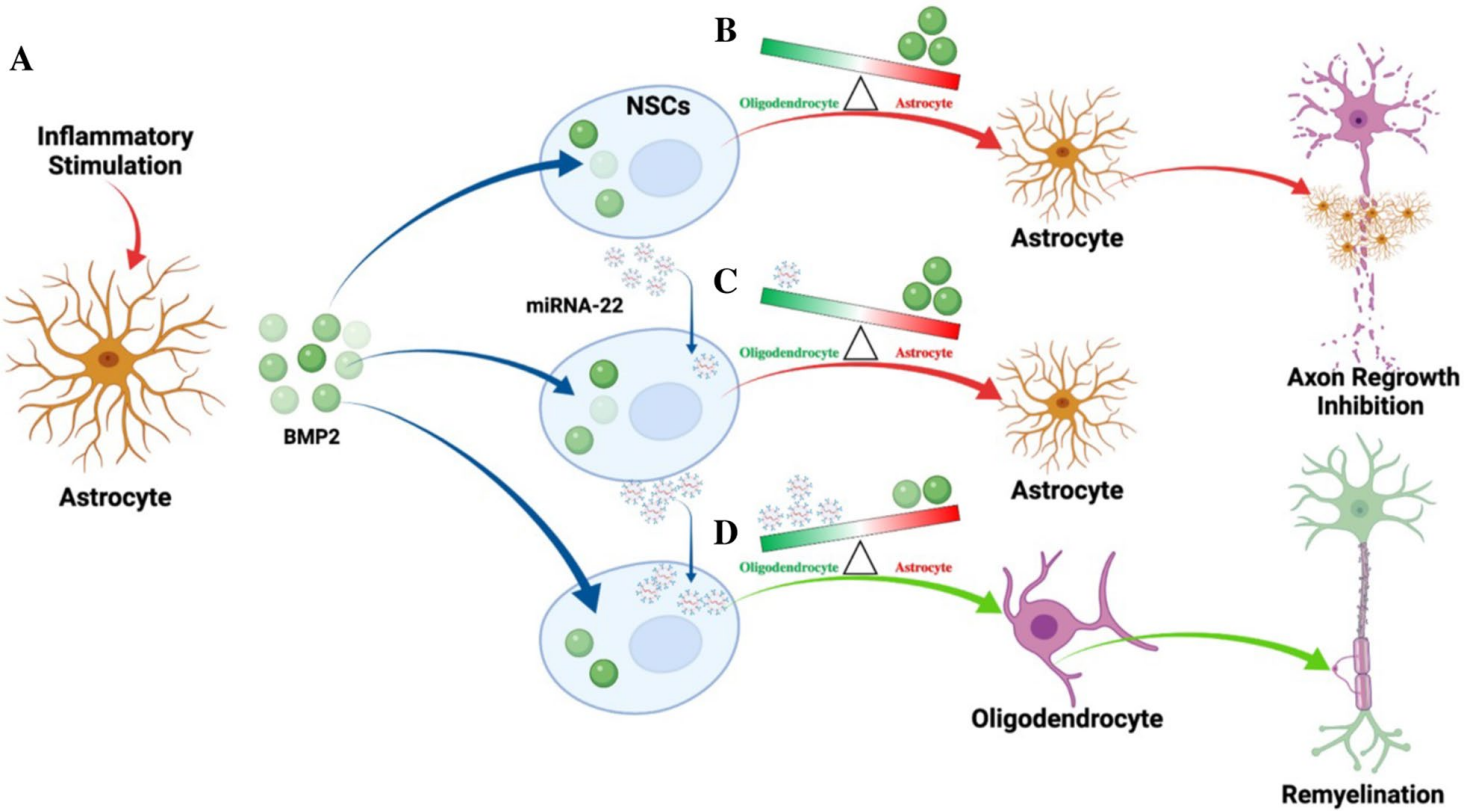
Astrocytes influence NSC differentiation and NSC–NSC communication. **A** Inflammation following SCI stimulated astrocytes to release more BMP2. **B** BMP2 promoted the differentiation of NSCs into astrocytes and enriches miRNA-22 within EVs derived from NSCs. **C** In the early phase of SCI, BMP2 accumulated around the cavity and remained at a relatively high level; it had a stronger effect on the regulation of NSC differentiation than miRNA-22. Therefore, in this period, NSCs differentiated into astrocytes, forming astrocytic barriers limiting the spread of inflammation. **D** With time, the expression of miRNA-22 increased with the decrease in BMP2, and the BMP2-induced effect in regulating the differentiation of NSCs weakened gradually. When the BMP2-induced effect was weaker than that of miRNA-22, NSCs differentiated into oligodendrocytes, promoting axon regrowth and remyelination

In addition, the results of culturing NSCs with different concentrations of LPS-As-CM and LPS-As-NSC-EVs showed that with the reduction of the dose of the LPS-As-CM, the differentiation of NSCs switched from astrocytes to oligodendrocytes. It indicated that in the early phase, as inflammatory cytokines rapidly immigrate and accumulated into the injured lesions following SCI, the astrocyte–NSCs communication (by releasing BMPs) takes the dominant role in mediating the differentiation of NSCs, causing most of the NSCs differentiate into astrocytes (Fig. 10A–C). However, in the later phase, with the downregulation of the inflammation and the BMPs within the astrocyte–NSCs communication, the NSC–NSC (by releasing miR-22) communication might take the dominant role, leading the differentiation of NSCs into more oligodendrocytes (Fig. 10D). Therefore, we thought that the final outcome of the differentiation of NSCs was determined by not the astrocyte–NSCs communication or NSCs–NSCs communication alone, but by the balance of astrocytes–NSCs communication and the NSCs–NSCs communication (Fig. 10).

## Conclusions

Astrocytes work as a bridge connecting inflammation and the regeneration of axons. Inflammation following SCI triggered astrocytes to release more BMP2, which, in turn, promoted the differentiation of endogenous NSCs into astrocytes, forming the glial boundary and limiting the spread of destructive inflammation. The astrocyte-released BMP2 upregulated miRNA-22 within NSCs-EVs, which promoted the differentiation of the adjacent NSCs into oligodendrocytes by targeting the KDM3A/TGF-β axis. As the effects of miRNA-22 were stronger than those of TGF-β on the differentiation of NSCs, these NSCs started to differentiate into oligodendrocytes, leading to axonal regrowth and remyelination into the glial boundary.

## Supplementary Information

**Additional file 1**.**Figure S1**: A. Identification of BMSC-EVs by transmission electron microscopy. B. Analysis of CD9, CD63, and TSG101 expression by western blot C. Detection of the diameter of BMSC-EVs by dynamic light scattering.

**Additional file 2. Figure S2:** The full-length of original gels.

**Additional file 3**. **Figure S3**: LPS-As-CM addition significantly upregulated the expression of miRNA-22 within NSC-EVs, which could inhibit the expression of KDM3A in NSCs. A. the expressions of axon-related and remyelination-related miRNAs within As-NSC-EVs and LPS-As-NSC-EVs were detected by PCR (n = 3; data are the mean ± S.D.; *p < 0.05, # p>0.05). B. the expression of the predicted genes was detected in NSCs by PCR with or without transfection of the miRNA-22 mimics (n = 3; data are the mean ± S.D.; *p < 0.05, # p>0.05). C. Dual luciferase reporter analysis showed that the luciferase activity was not altered by the transfection of miRNA-22 mimics in the HOMER1 and TGFBR1 groups (n = 3; data are the mean ± S.D.; *p < 0.05, # p>0.05).

## Data Availability

The datasets used and/or analyzed during the current study are available from the corresponding author upon reasonable request.

## Abbreviations

SCI: Spinal cord injury
NSCs: Neural stem cells
EVs: Extracellular vesicles
BMPs: Bone morphogenetic proteins
CM: Conditioned medium
GFAP: Glial fibrillary acidic protein
TGF-β: Transforming growth factor β
TUJ-1: Neuron-specific class III beta-tubulin
p75: p75 neurotrophin receptor
Sox10: SRY-Box transcription factor 10
cnpase: 2′3′ Cyclic nucleotide 3′ phosphodiesterase
CNS: Central nervous system
LPS: Lipopolysaccharide
IL-1α: Interleukin-1 alpha
TNF-α: Tumor necrosis factor alpha
