# Supplementary figures and images for: Inflammatory stimulation of astrocytes affects the expression of miRNA-22-3p within NSCs-EVs regulating remyelination by targeting KDM3A
Source: Stem Cell Res Ther. 2023 Mar 23;14:52. doi: 10.1186/s13287-023-03284-w (PMC10035185; doi:10.1186/s13287-023-03284-w)

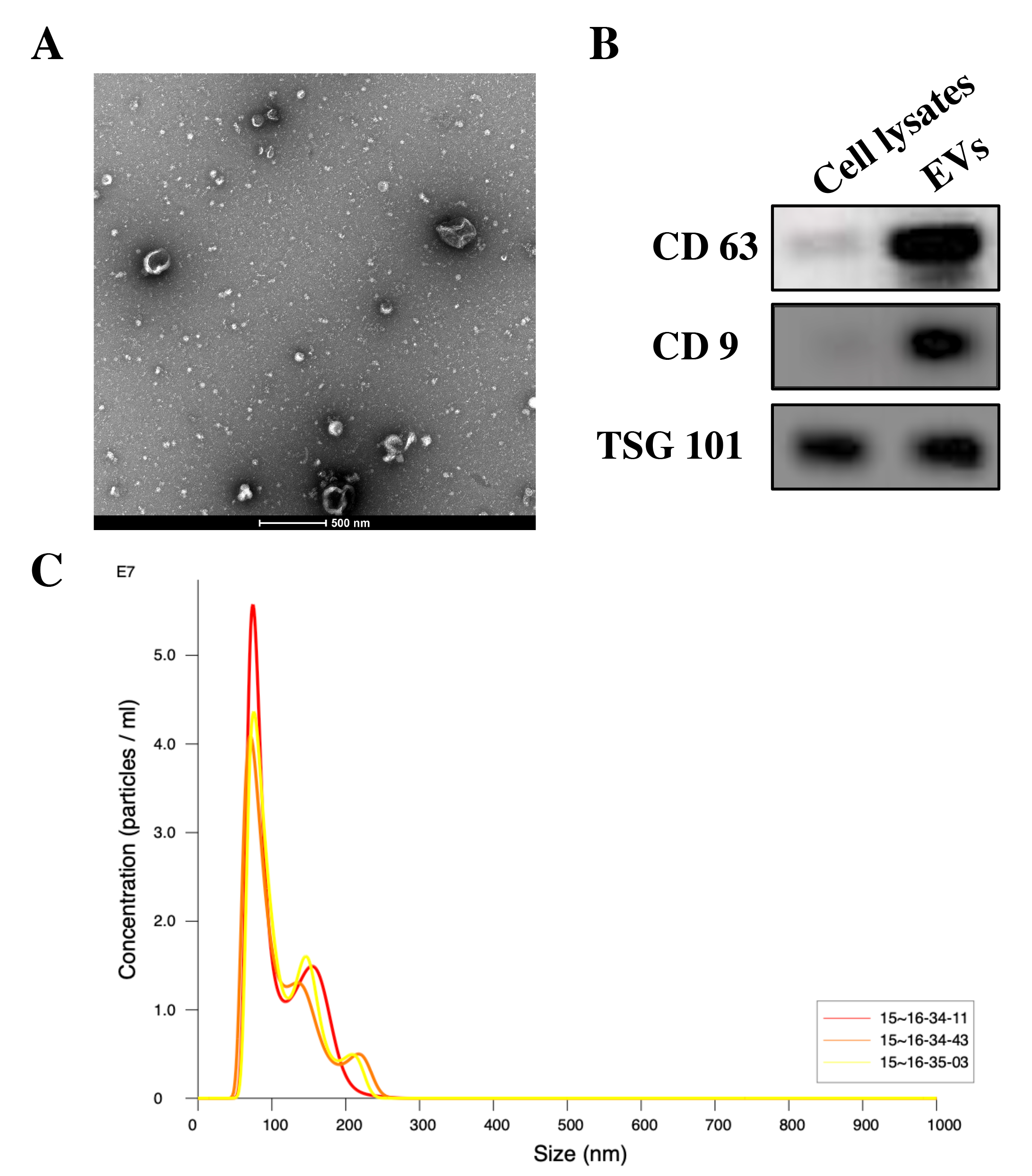

Supplement: Supplementary file 1 — Additional file 1.Figure S1: A. Identification of BMSC-EVs by transmission electron microscopy. B. Analysis of CD9, CD63, and TSG101 expression by western blot C. Detection of the diameter of BMSC-EVs by dynamic light scattering. [file 13287_2023_3284_MOESM1_ESM.tif]

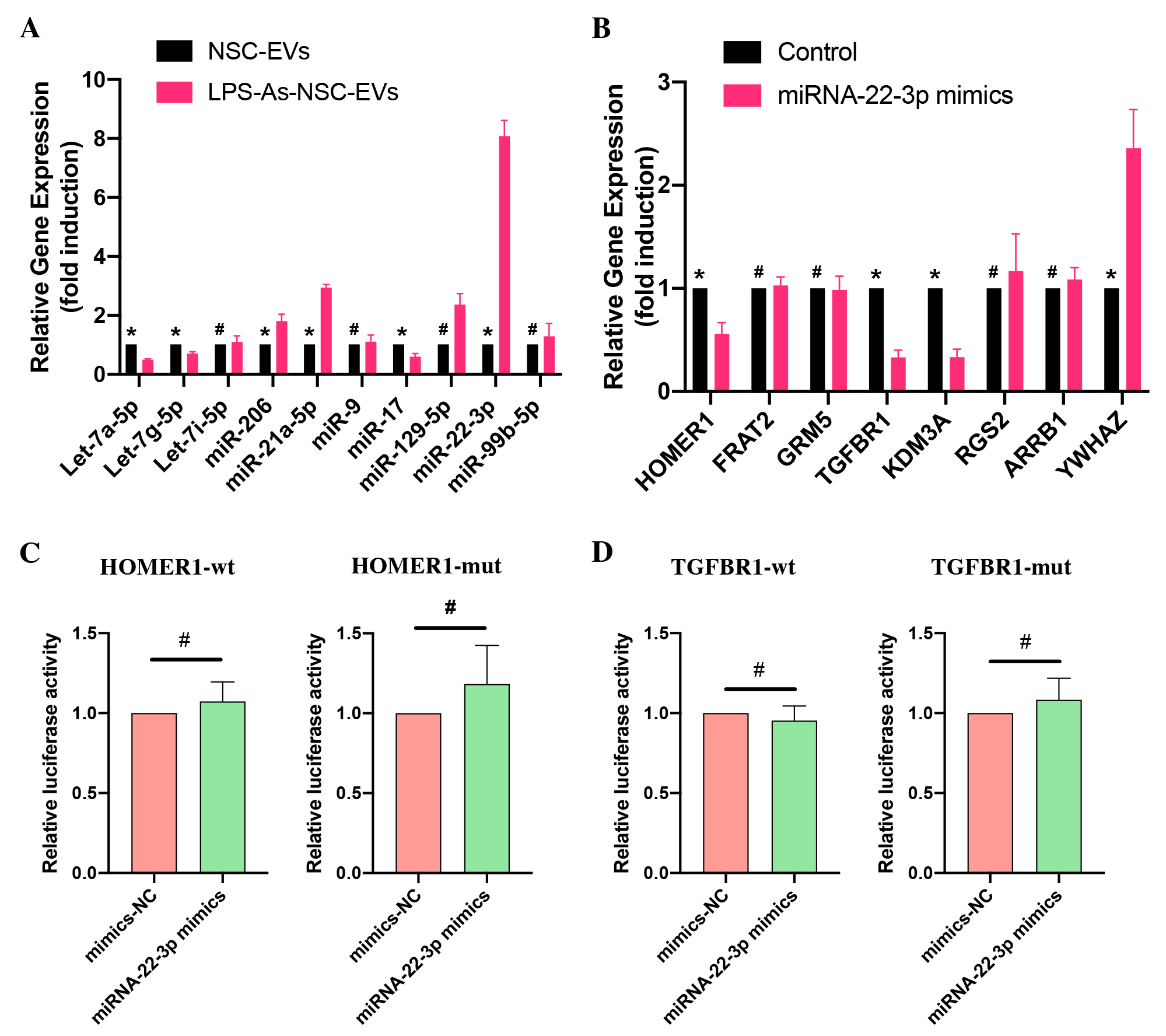

Supplement: Supplementary file 3 — Additional file 3. Figure S3: LPS-As-CM addition significantly upregulated the expression of miRNA-22 within NSC-EVs, which could inhibit the expression of KDM3A in NSCs. A. the expressions of axon-related and remyelination-related miRNAs within As-NSC-EVs and LPS-As-NSC-EVs were detected by PCR (n = 3; data are the mean ± S.D.; *p < 0.05, # p>0.05). B. the expression of the predicted genes was detected in NSCs by PCR with or without transfection of the miRNA-22 mimics (n = 3; data are the mean ± S.D.; *p < 0.05, # p>0.05). C. Dual luciferase reporter analysis showed that the luciferase activity was not altered by the transfection of miRNA-22 mimics in the HOMER1 and TGFBR1 groups (n = 3; data are the mean ± S.D.; *p < 0.05, # p>0.05). [file 13287_2023_3284_MOESM3_ESM.tif]
