# Supplementary material for: Inflammatory stimulation of astrocytes affects the expression of miRNA-22-3p within NSCs-EVs regulating remyelination by targeting KDM3A
Source: Stem Cell Res Ther. 2023 Mar 23;14:52. doi: 10.1186/s13287-023-03284-w (PMC10035185; doi:10.1186/s13287-023-03284-w)
Supplement: Supplementary file 2 — Additional file 2. Figure S2: The full-length of original gels. [file 13287_2023_3284_MOESM2_ESM.pdf]

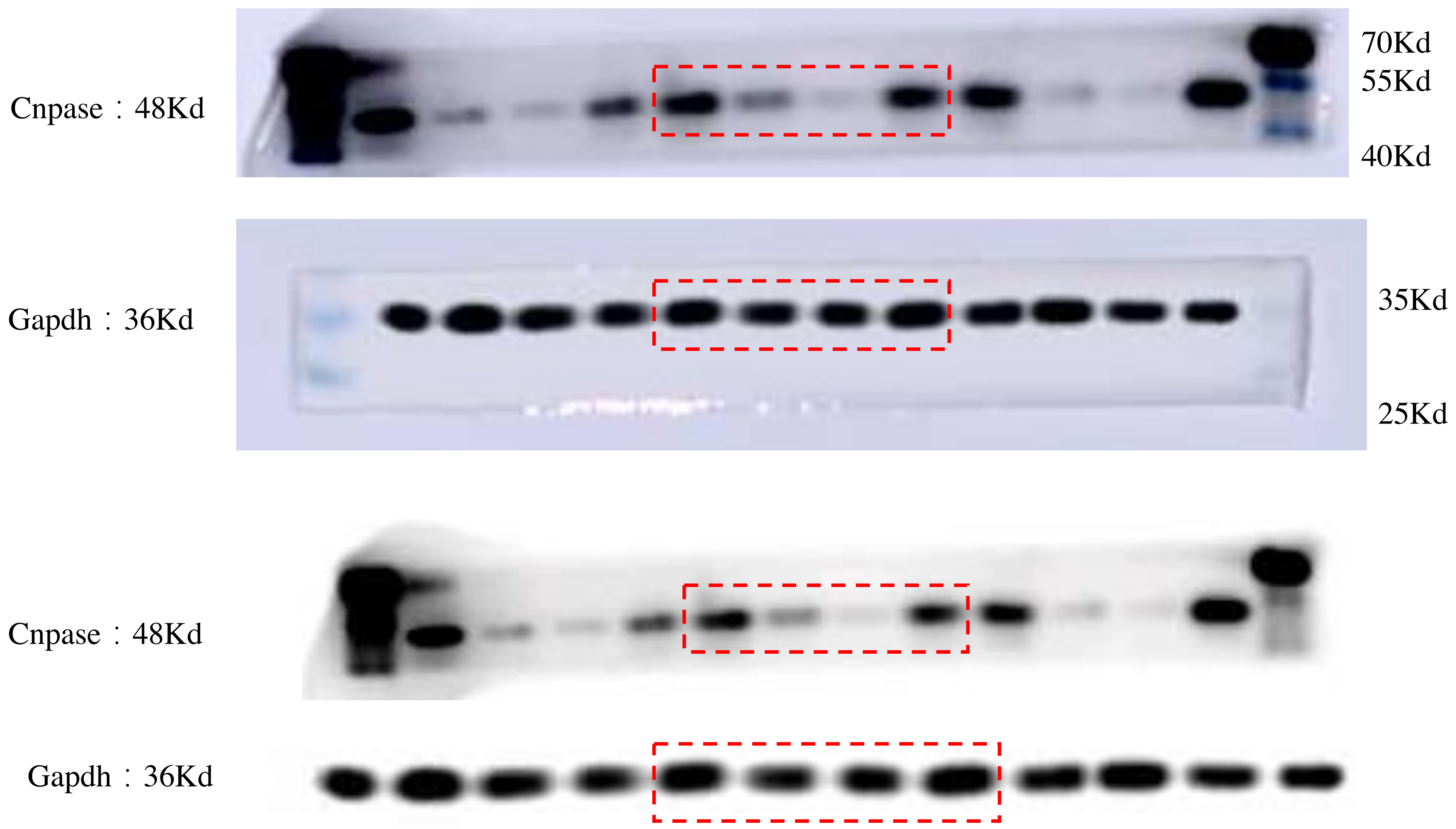

The Full-length blots/gels for Figure.2 F

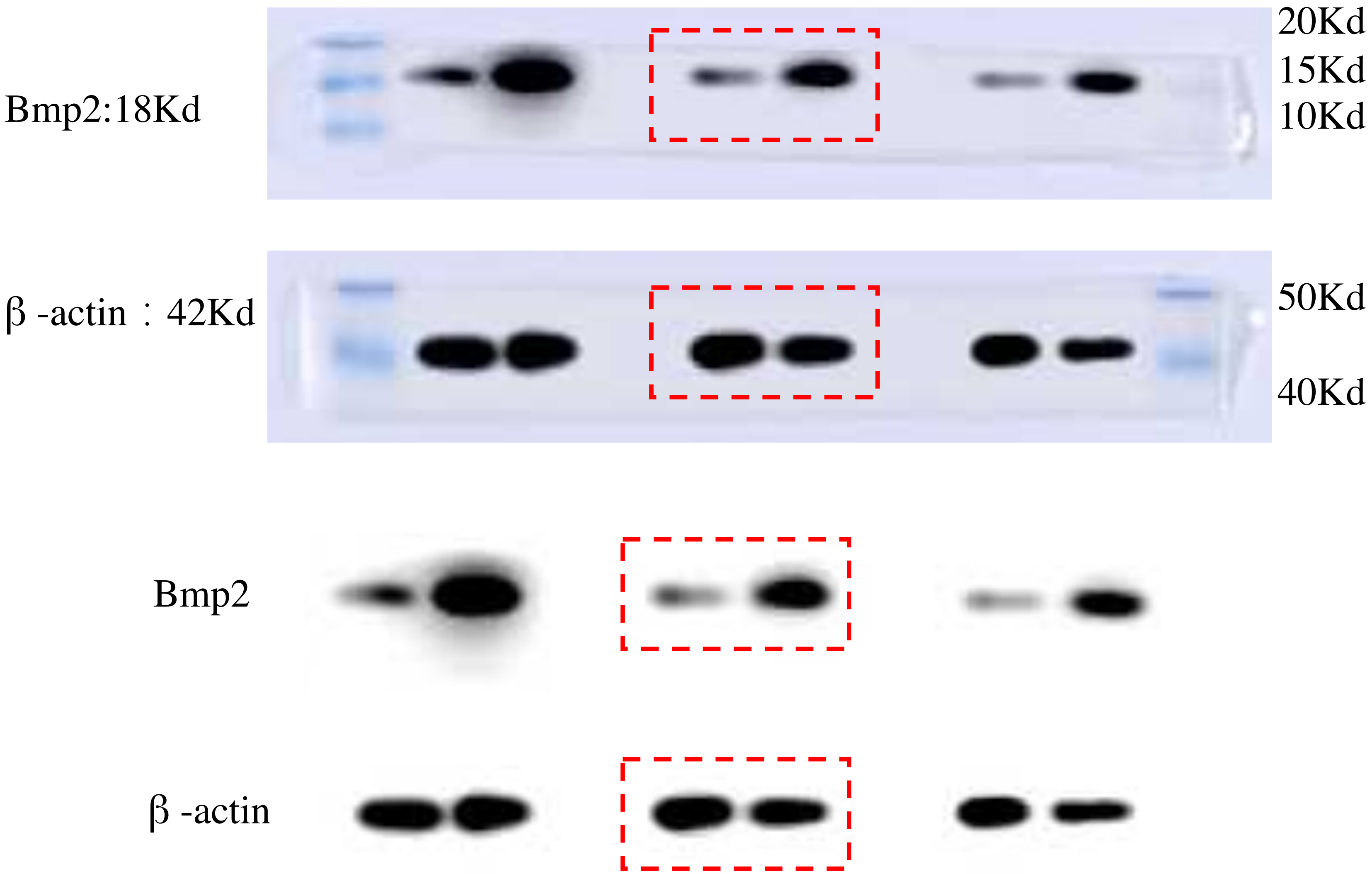

The Full-length blots/gels for Figure.3 B

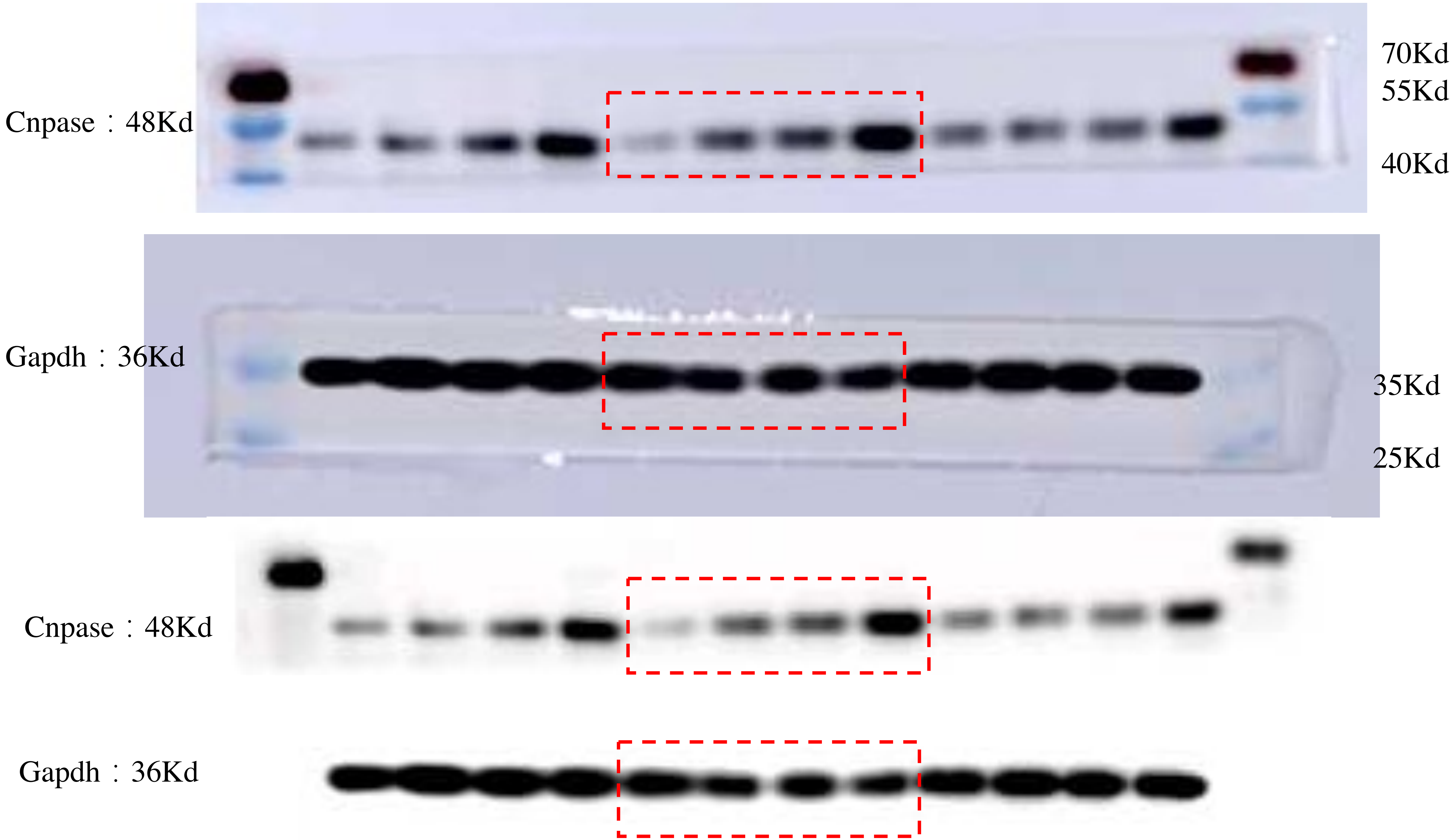

The Full-length blots/gels for Figure.5 E

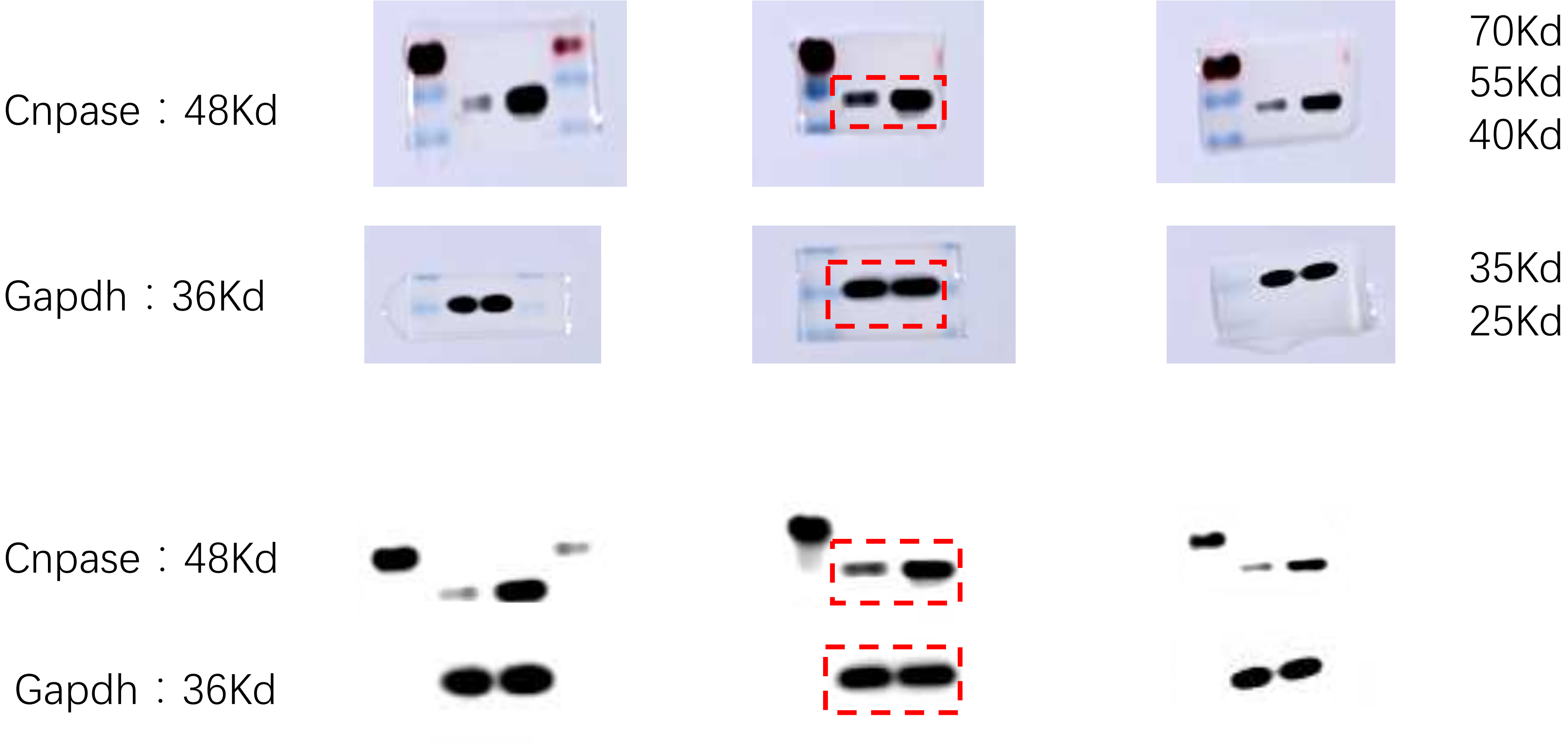

The Full-length blots/gels for Figure.7 J

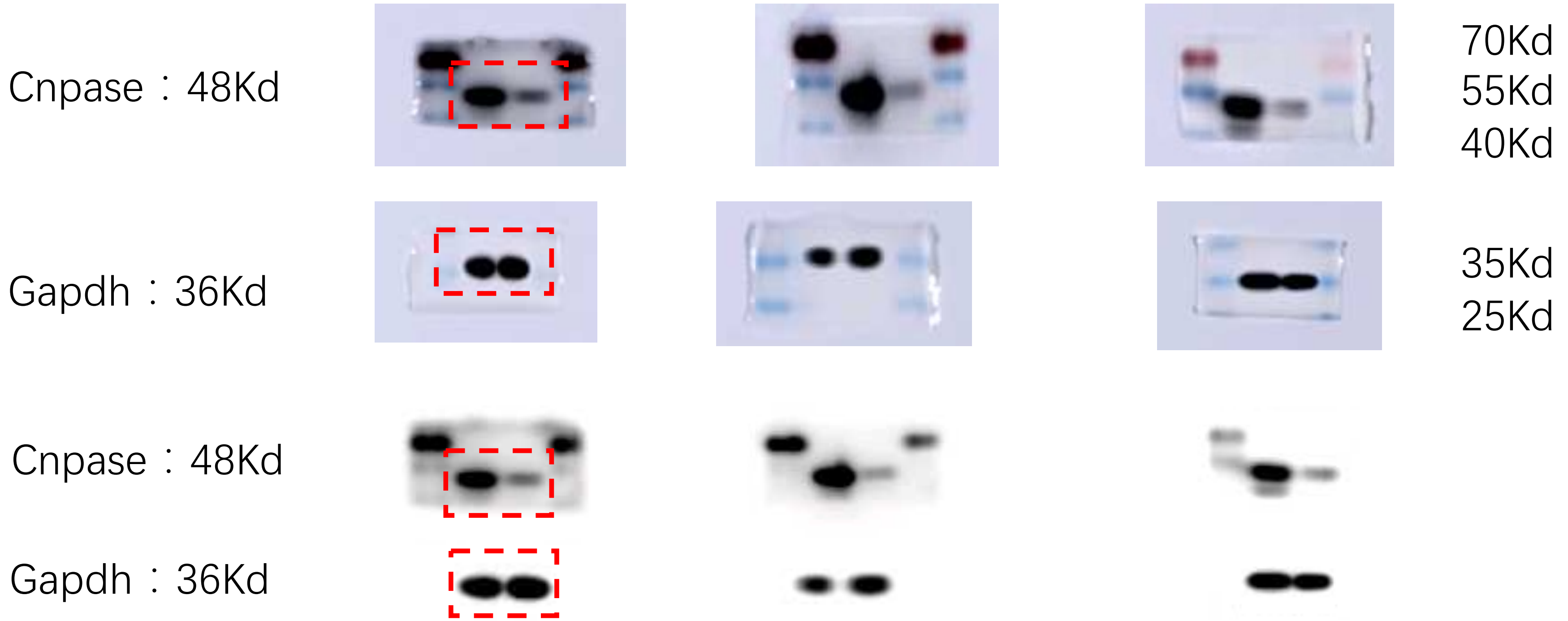

The Full-length blots/gels for Figure.8 D

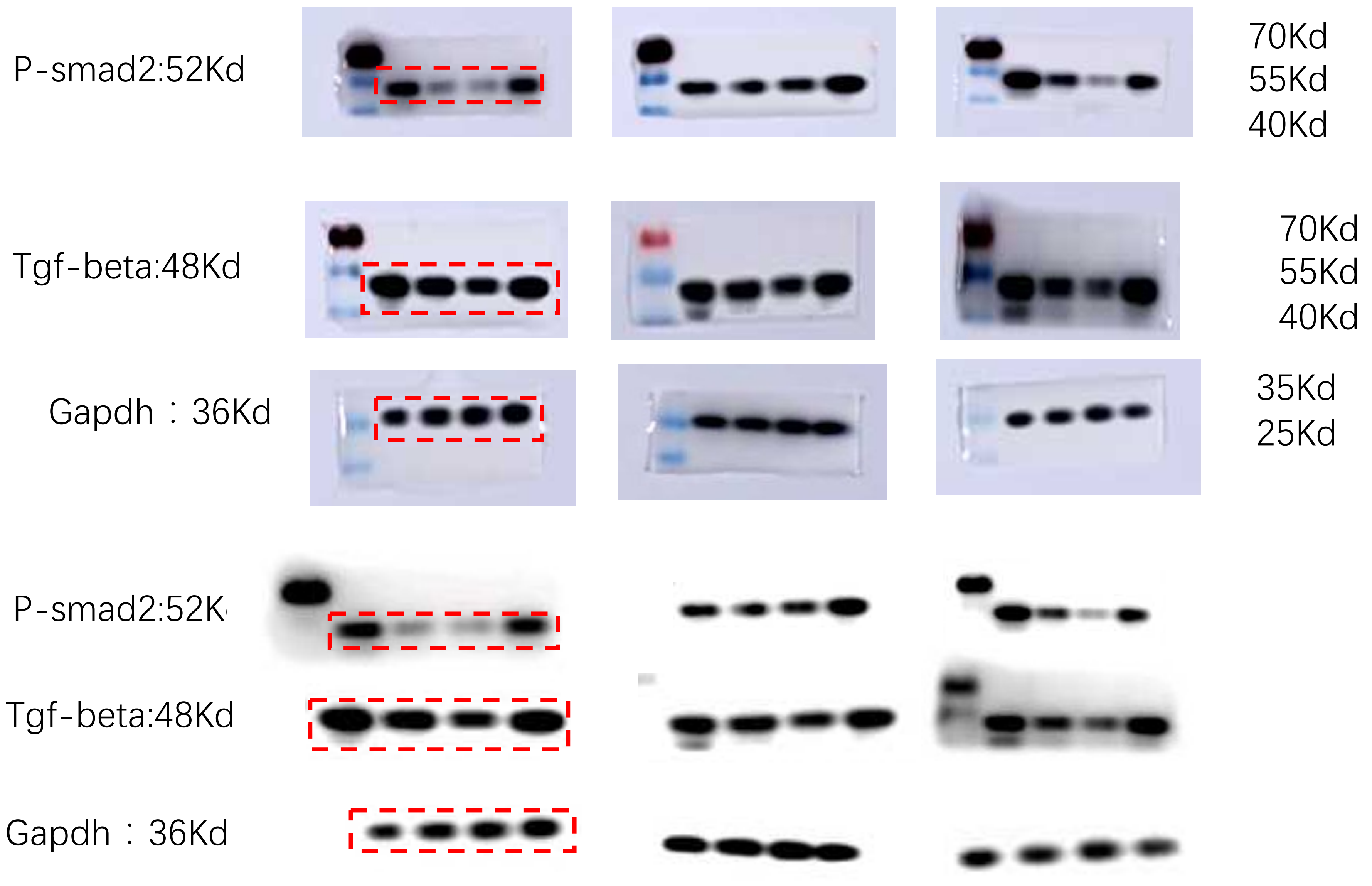

The Full-length blots/gels for Figure.9 D
